# TET2 and TET3 loss disrupts small intestine differentiation and homeostasis

**DOI:** 10.1038/s41467-023-39512-3

**Published:** 2023-07-06

**Authors:** Ihab Ansari, Llorenç Solé-Boldo, Meshi Ridnik, Julian Gutekunst, Oliver Gilliam, Maria Korshko, Timur Liwinski, Birgit Jickeli, Noa Weinberg-Corem, Michal Shoshkes-Carmel, Eli Pikarsky, Eran Elinav, Frank Lyko, Yehudit Bergman

**Affiliations:** 1 Department of Developmental Biology and Cancer Research, Institute for Medical Research Israel-Canada, Hebrew University Medical School, Jerusalem, Israel; 2 Division of Epigenetics, DKFZ-ZMBH Alliance, German Cancer Research Center, Heidelberg, Germany; 3 Department of Immunology, The Weizmann Institute of Science, Rehovot, Israel; 4 University Psychiatric Clinics Basel, Clinic for Adults, University of Basel, Basel, Switzerland; 5 The Lautenberg Center for Immunology, Institute for Medical Research Israel-Canada, Hebrew University Medical School, Jerusalem, Israel; 6 Division of Microbiome and Cancer, German Cancer Research Center (DKFZ), Heidelberg, Germany

**Keywords:** Differentiation, Epigenetics, Microbiome, Small intestine

## Abstract

TET2/3 play a well-known role in epigenetic regulation and mouse development. However, their function in cellular differentiation and tissue homeostasis remains poorly understood. Here we show that ablation of TET2/3 in intestinal epithelial cells results in a murine phenotype characterized by a severe homeostasis imbalance in the small intestine. *Tet2/3*-deleted mice show a pronounced loss of mature Paneth cells as well as fewer Tuft and more Enteroendocrine cells. Further results show major changes in DNA methylation at putative enhancers, which are associated with cell fate-determining transcription factors and functional effector genes. Notably, pharmacological inhibition of DNA methylation partially rescues the methylation and cellular defects. TET2/3 loss also alters the microbiome, predisposing the intestine to inflammation under homeostatic conditions and acute inflammation-induced death. Together, our results uncover previously unrecognized critical roles for DNA demethylation, possibly occurring subsequently to chromatin opening during intestinal development, culminating in the establishment of normal intestinal crypts.

## Abstract (web summary)

DNA demethylation is known to be critical for the development and function of many tissues. Here the authors show that it is also required for intestinal lineage differentiation, and that mice lacking DNA demethylases have altered microbiomes and a predisposition to inflammation.

## Introduction

The mammalian intestine is renewed every 4–5 days. Intestinal stem cells (ISCs), which reside in the crypts, continuously proliferate to fuel the high turnover of the intestinal epithelium^1^. ISCs divide asymmetrically, giving rise to additional stem cells or to progenitor cells (absorptive and secretory) that undergo a limited number of cell divisions before terminal differentiation. Absorptive progenitors differentiate into enterocytes, which represent the most abundant cell type in the intestinal epithelium, whereas secretory progenitors give rise to Goblet, Paneth, Enteroendocrine (EE), and Tuft cells^2^. Together, these distinct cell types cooperate to facilitate effective nutrient absorption while also providing an essential barrier that protects the gut from toxic substances and pathogens.

DNA methylation occurs in a highly regulated manner during normal embryonic development^3–5^. Once the methylome is established, genomic regions can undergo developmentally programmed and directed changes^6^. Tissue-specific DNA methylation changes include de novo methylation and demethylation^7,8^, which are catalyzed by DNMTs and the TET family of enzymes, respectively^9^. The role of TET enzymes (TET1, 2, 3) has been extensively studied in several developmental tissues, most notably in the hematopoietic system^10–12^. We have previously shown that loss of TET2/3 in the early stages of B-cell development largely prevents lineage-specific programmed demethylation events, culminating in an abnormal B-cell differentiation in vivo^13^. Additionally, it has been shown that loss of TET2/3 in T regulatory cells induces inflammatory diseases^14^. Thus, tissue-specific DNA demethylation appears to be necessary for proper somatic cell development as well as for regulating the physiological cell state. However, the precise role of TET2/3 in intestinal development under homeostatic conditions remains largely undefined.

Intestinal development in mice undergoes various transitional stages, ranging from embryonic day (E)14 until early postnatal life (postnatal day P15). The postnatal period is critical for the proper maturation of the intestine in response to microbial colonization, oral nutrition, weaning, and nutritional availability^15–18^. Changes in both gene expression and DNA methylation patterns^19–22^ were observed throughout the transitions between fetal, suckling, weaning, and adult life periods^16,19^. None of these studies, however, has addressed the key question of whether demethylation itself is actually required for proper intestinal lineage differentiation, thereby affecting the capability of the intestine to sustain a normal microbiome.

Recently, we have explored the impact of the gut microbiota on DNA methylation in the mouse colon under normal homeostasis and acute inflammation, demonstrating that the microbiota induces TET2/3-mediated DNA demethylation at regulatory elements^23^. Here we show that TET2/3 loss in the small intestine (SI), resulted in a massive loss of mature Paneth cells, reduced Tuft cell, and increased EE cell numbers. Integration of our genome-wide analyses revealed that loss of TET2/3 resulted in hypermethylation of thousands of regulatory elements required for proper cellular differentiation that can be partially restored using pharmacological inhibition of DNA methylation in vivo. Moreover, the loss of TET2/3 resulted in an altered microbiome, presumably increasing intestinal susceptibility to inflammatory signals.

## Results

### TET2/3-deletion alters intestinal homeostasis

**Fig. 1:**
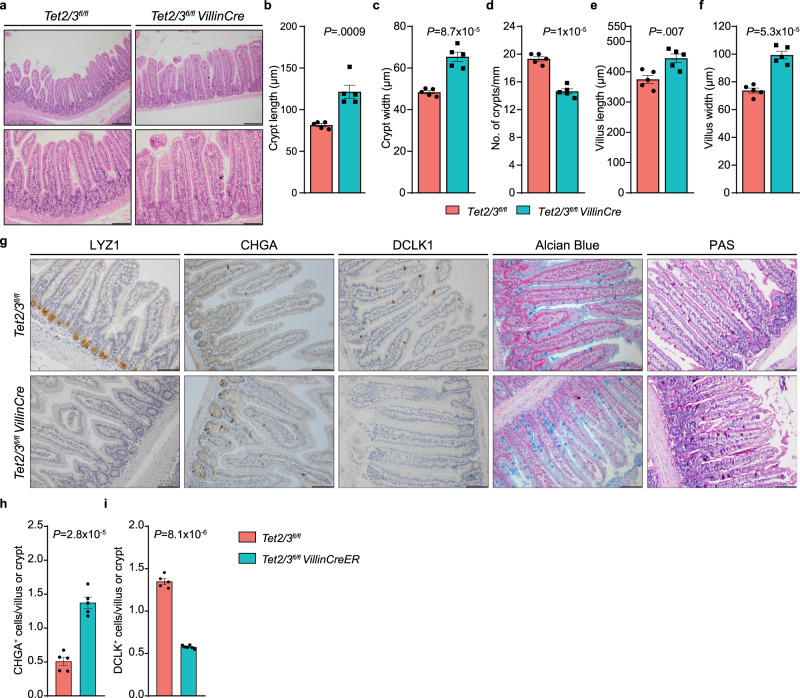
TET2/3 play a role in SI homeostasis. **a** Hematoxylin and Eosin (H&E)-stained histologic images of the jejunum from *Tet2/3*^*fl/fl*^ (wt) and *Tet2/3*^*fl/fl*^ *VillinCre* (dko) mice. Scale bar 100 µm (upper) and 50 µm (lower). **b**, **c** Statistical analysis of crypt length (**b**) and crypt width (**c**) of wt (*n* = 5) and dko (*n* = 5) SI. **d** Statistical analysis of crypts number in wt (*n* = 5) and dko (*n* = 5) SI-jejunum. Statistical analysis of villus length (**e**) and villus width (**f**) of wt (*n* = 5) and dko (*n* = 5) SI. **g** Immunohistochemical staining for LYZ1, CHGA, DCLK1, Alcian Blue (AB) and periodic acid-schiff stain (PAS) of the SI from dko mice, compared with wt mice. Scale bar 50 µm. **h** Quantification of CHGA -positive cells in wt (*n* = 5) and dko (*n* = 5) mice. **i** Quantification of DCLK1-positive cells in wt (*n* = 5) and dko (*n* = 6) mice. Significance (**b**–**i**) was determined using a two-sided *t*-test and is expressed as the mean ± SEM. Source data are provided as a Source Data file.

To understand the role of TET2 and TET3 in the SI, we generated intestinal-specific TET2/3 double knock-out (dko) mice by crossing *Tet2/3*^*fl/fl*^ (wt) with *VillinCre* mice. Deletion of both *Tet2* and *Tet3* in the intestine was confirmed by genotyping and mRNA level (Supp. Fig. 1a–e). To explore SI homeostasis in dko mice, we first carried out a stringent histological analysis. The results revealed longer and wider villi and crypts in the dko mice when compared with the wt mice (Fig. 1a–f). To decipher the effects of TET2/3 loss on the cell composition of the SI, we performed immune-histological analyses with specific antibodies directed against different cell type markers in the jejunum (Fig. 1g–i), as well as in the duodenum, ileum, and colon (Supp. Fig. 1f–l). Strikingly, antibodies directed against LYZ1, a Paneth cell marker, hardly revealed any signal in dko mice (Fig. 1g and Supp. Fig. 1f), indicating a robust decrease of this cell type upon TET2/3 depletion. Of note, the Paneth cells loss was observed in both sexes and in young (P14) mice (a time point in which around 50% of the total Paneth cells exist under normal conditions^24^), but not in single knockouts of either TET2 or TET3 (Supp. Fig. 2a–d), indicating that both enzymes are required for the aberrant phenotype. Moreover, our results also showed that the number of EE cells, highlighted with anti-CHGA immunostaining, increased in the dko jejunum by about threefold, while the number of Tuft cells, identified with anti-DCLK1, decreased by more than 50% (Fig. 1g–i). The Tuft cells are an epithelial cell type critical for type 2 immune responses to parasites and protozoa. Staining of Goblet cells with Alcian Blue (AB) and Periodic Acid-Schiff stain (PAS), did not show evident differences between wt and dko jejunum (Fig. 1g). Similar phenotypes were also observed in the rest of the SI and in the colon (Supp. Fig. 1f–l).

**Fig. 2:**
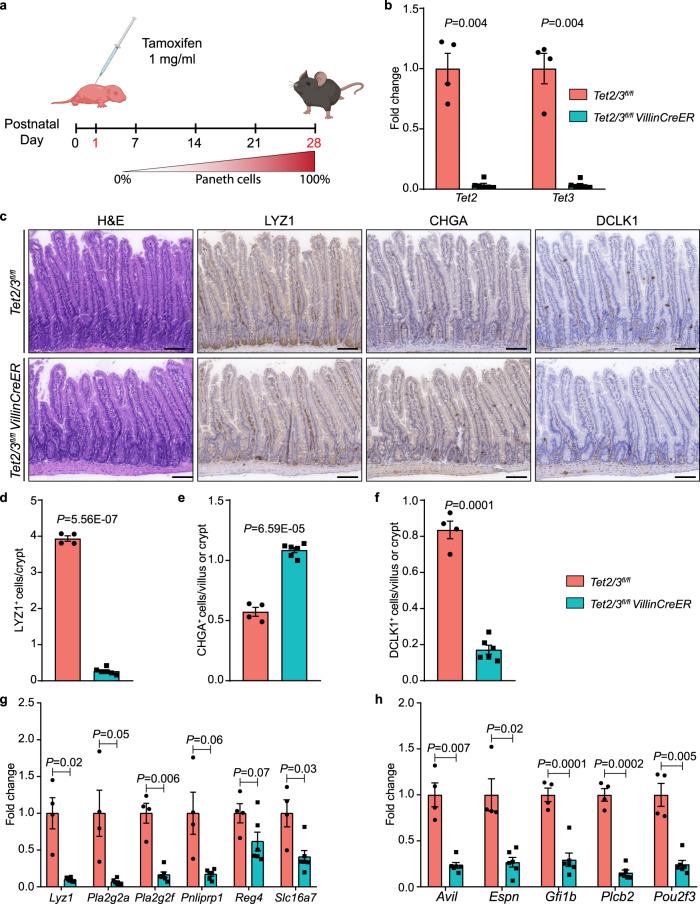
TET2/3 activity is required postnatally. **a** Schematic diagram of the experimental design. On postnatal day 1 (P1) males and females pups were intragastric injected with 1 mg/ml tamoxifen. On P28 the mice were sacrificed, and the intestinal crypts from the jejunum region were used for further analyses. The schematic was created using BioRender.com. **b** Normalized expression levels of *Tet2* and *Tet3* genes from SI crypts isolated from *Tet2/3*^*fl/fl*^ (wt) (*n* = 4) and *Tet2/3*^*fl/fl*^ *VillinCreER* (dko) (*n* = 6) mice treated with tamoxifen. **c** H&E, LYZ1-, CHGA-, and DCLK1- stained histologic images of the jejunum from wt and dko mice, treated with tamoxifen. Scale bar 100 µm. **d** Quantification of LYZ1-positive cells in wt (*n* = 4) and dko (*n* = 6) mice. **e** Quantification of CHGA-positive cells in wt (*n* = 4) and dko (*n* = 6) mice. **f** Quantification of DCLK1-positive cells in wt (*n* = 4) and dko (*n* = 6) mice. **g** Expression levels of several Paneth cell markers in wt (*n* = 4) and dko mice (*n* = 6) treated with tamoxifen. **h** Expression levels of several Tuft markers in wt (*n* = 4) and dko mice (*n* = 6) treated with tamoxifen. Significance (**b**, **d**–**h**) was determined using a two-sided *t*-test and is expressed as the mean ± SEM. Source data are provided as a Source Data file.

Given the above-described phenotype, we next asked whether TET2/3 are required during embryonic development or only after birth. To this end, we crossed *Tet2/3*^*fl/fl*^ with *VillinCreER* mice to generate TET2/3 inducible mutant mice^25^. Postnatal mice (P1) were injected once with tamoxifen (TAM) (Fig. 2a) to induce TET2/3-deletion, and at P28 mice were sacrificed and SI crypts were isolated and analyzed to verify *Tet2/*3-deletion (Fig. 2b). Immune-histological analyses with specific antibodies directed against different cell type markers in the jejunum revealed a comparable phenotype to the constitutive dko mice, with a massive loss of mature Paneth cells concomitant with reduced Tuft and increased EE cells (Fig. 2c–f). In addition, qPCR analyses of Paneth and Tuft cell-type specific markers further validated the phenotype of the inducible TET2/3 dko (Fig. 2g, h). These findings indicate that TET2/3 activity is required postnatally rather than during embryonic development.

### TET2/3-deletion causes altered differentiation trajectories

**Fig. 3:**
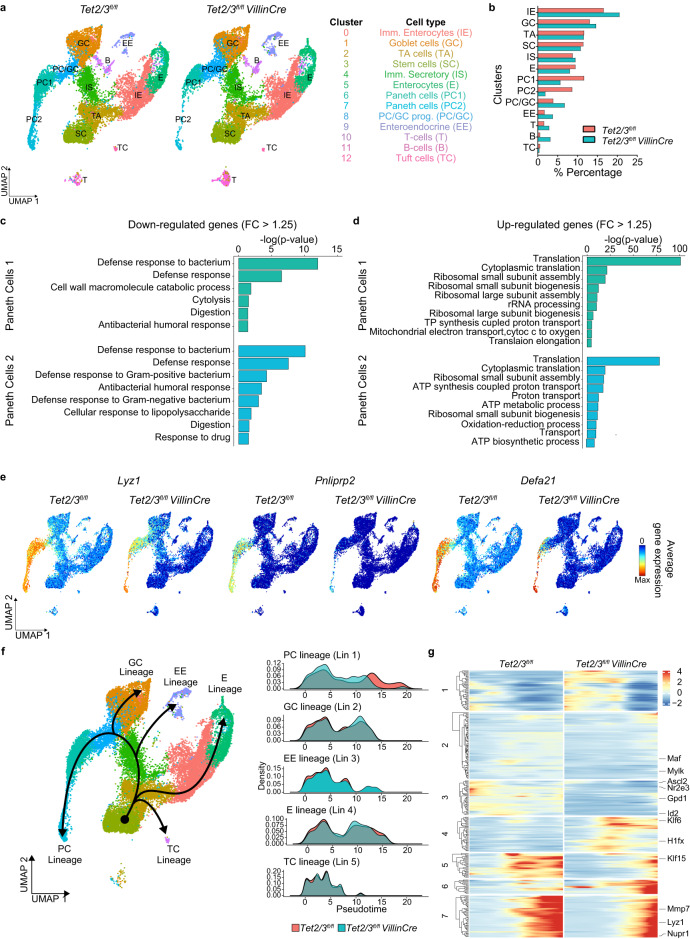
Single-cell RNA sequencing analysis of *Tet2/3*^*fl/fl*^ and *Tet2/3*^*fl/fl*^*VillinCre* SI. **a** UMAP visualization of unsupervised clustering of identified 13 distinct intestinal cell clusters in the jejunum of wt and dko crypts. **b** Bar plot indicates the percentage of intestinal cells corresponding to each subpopulation in the SI of wt and dko mice. **c** Gene ontology (GO) analysis of the downregulated genes in Paneth cell clusters (PC1 and PC2) of dko compared to wt samples. The highly enriched processes are shown (*P* values were calculated using two-tailed Fisher’s exact test). **d** Gene Ontology (GO) analysis of the upregulated genes in Paneth cell clusters (PC1 and PC2) of dko compared to wt samples. The highly enriched processes are shown (*P* values were calculated using two-tailed Fisher’s exact test). **e** UMAP visualization showing the expression of Paneth-related genes *Lyz1*, *Pnliprp2*, and *Defa21* in each cluster of wt and dko samples. In all UMAP gene expression projections, red indicates maximum gene expression while blue indicates low or no expression in log-normalized unique molecular identifier (UMI) counts. **f** Two-dimensional representation of scRNA-seq data using UMAP (left panel). The intestinal trajectory inferred by slingshot is displayed (Lin1: PC lineage, Lin2: GC lineage, Lin3: EE lineage, Lin4: enterocytes (E) lineage and Lin5: Tuft cell (TC) lineage). Superimposed pseudotime densities (right panel) for each lineage in wt and dko samples. Only few cells of linage 1 in the dko mice managed to properly differentiate. **g** Heatmaps showing the expression patterns of the differentially expressed genes along pseudotime between wt and dko samples for the PC lineage. Transcription factors and Paneth cell gene markers are highlighted. Source data are provided as a Source Data file.

To gain more insight into the SI phenotype of TET2/3-deficient mice, we performed single-cell RNA sequencing (scRNA-seq) on isolated crypts from three independent 8–10 weeks old wt and constitutive dko mice. To avoid confounding factors, all samples were obtained from the jejunum. In an initial analysis, we obtained an overview of the diverse intestinal populations by integrating the cells from all six samples. Data analysis of the 25,693 cells that passed our quality control identified 13 clusters with distinct expression profiles (Supp. Table 1 and Supp. Fig. 3a), which were visualized in a uniform manifold approximation and projection (UMAP) plot (see Methods)^26^. Importantly, all identified clusters contained cells from all animals, regardless of their genotype (Supp. Fig. 3a). Comparing known markers with the most representative expressed genes of each cluster (Supp. Fig. 3b–d) revealed the identity of the 13 cell clusters (see Methods) (Fig. 3a). Importantly, significant changes in the proportions of various cell populations were observed in dko intestines (Fig. 3a, b). In line with our previous results, we noticed a decrease in Tuft cells (cluster 12, TC), as well as an increase in EE cell (cluster 9) (Fig. 3b). The EE compartment is known to contain several subtypes with distinct functions^27,28^. Based on the expression of important developmental genes and hormone genes we could identify EE subtypes similar to those previously described in ref. ^27^ (Supp. Fig. 4a, b). Of note, while we observed a general increase in EE cells in dko mice, our refined analysis revealed a decreased proportion of EE progenitors and an increased proportion of X-cells (Ghrl) and L-cells (Gcg) in the dko as compared to wt mice (Supp. Fig. 4a–d).

Importantly, the most pronounced difference between wt and dko mice was found in Paneth cells, which were represented by two clusters (clusters 6 and 7 or PC1 and PC2) and became strongly reduced in the dko mice (Fig. 3a, b). In subsequent analyses, we identified the differentially expressed genes between wt and dko cells in each population (Supp. Fig. 5a). Gene Ontology (GO) analyses of the downregulated genes in the PC1 and PC2 populations revealed a decreased expression of antibacterial genes in the dko mice, thus reflecting a loss of functional Paneth cells in dko mice (Fig. 3c). GO analyses of the upregulated genes indicated overexpression of ribosomal genes in the dko Paneth cells (Fig. 3d and Supp. Fig. 5b). The reduced numbers of Paneth cells and their loss of function in dko mice indicated by our scRNA-seq analyses was illustrated by the expression patterns of known Paneth cell markers (*Lyz1*, *Pnliprp2*, and *Defa21*), which were strongly downregulated in dko mice (Fig. 3e and Supp. Fig. 5c). In addition to their antimicrobial functions, Paneth cells also contribute to the stem cell niche^29^. To test whether this function is also affected by TET2/3 ablation, we generated organoids derived from wt and dko cells which could maintain the growth of stem cells in the absence of external Wnt ligands, regardless of the genotype (Supp. Fig. 5d). Thus, our data suggest that this function of Paneth cells is not majorly affected by the loss of TET2/3.

To further characterize epithelial differentiation in dko crypts, we performed trajectory inference analysis on our scRNA-seq dataset (excluding immune cells). Using wt and dko cells combined, this analysis identified five distinct epithelial cell lineages (Lin1–5) corresponding to the five major epithelial cell types found in the small intestine (Fig. 3f). These lineages recapitulated the expression patterns of well-known lineage-specific genes in both wt and dko samples (Supp. Fig. 6a). Nonetheless, further analyses highlighted the specific disruption of the Paneth cell lineage, suggesting a blockage in their differentiation in dko crypts, with only few cells reaching a differentiated state (Fig. 3f). Of note, 289 genes were found to be differentially expressed (FDR < 0.05) along the pseudotime of the Paneth cell lineage, including well-known genes for PC differentiation such as *Mmp7*, *Lyz1* and *Nupr1*, as well as transcription factors such as *Gpd1*, *Maf*, *Klf6* and *Klf15* (Fig. 3g). Of note, seven patterns of expression could be identified among the differentially expressed genes in PC lineage (Fig. 3g). Collectively, these results reveal that TET2/3 are required for proper intestinal homeostasis and suggest a differentiation block in the Paneth cell lineage upon their depletion.

### Paneth/Goblet progenitors are expanded in TET2/3-depleted crypts

**Fig. 4:**
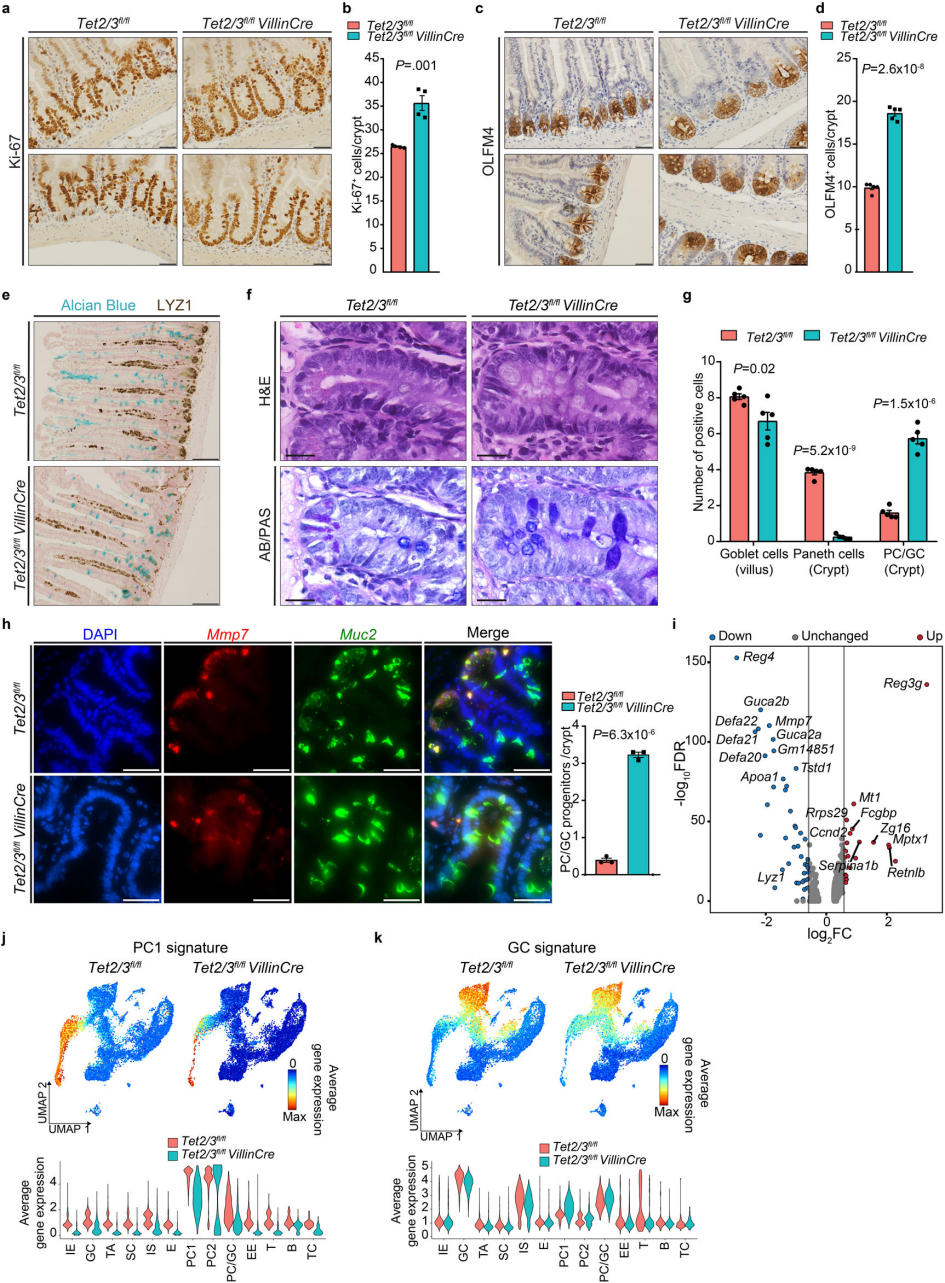
PC/GC progenitors are more abundant at the crypt base in dko mice. **a** Ki-67 staining on jejunum specimens from wt (*n* = 4) and dko (*n* = 4) mice. Scale bar 20 µm. **b** Quantification of Ki-67-positive cells. **c** OLFM4 staining on jejunum specimens from wt (*n* = 5) and dko (*n* = 5) mice. Scale bar 20 µm. **d** Quantification of OLFM4-positive cells. **e** Double staining for LYZ1 (brown) and Alcian Blue (blue) on jejunum specimens from wt (*n* = 3) and dko mice (*n* = 3). Scale bar 50 µm. **f** H&E and Alcian Blue/PAS staining of jejunum specimens from wt (*n* = 5) and dko (*n* = 5) mice. Scale bar 20 µm. **g** Quantitation of Paneth, goblet and PC/GC cells in the crypts and villi. At least 50 crypts and villi were counted from each mouse of each genotype (*n* = 5). **h** Cryo-sections of mouse SI stained for DNA (DAPI, blue) and hybridized with smFISH probes against *Mmp7* (red) and *Muc2* (green). Since Paneth cells are known to express low amounts of Muc2^101^, we assigned as Goblet-like cells, cells that show Goblet morphology harboring an intense staining for *Muc2*. Scale bar 50 µm. Quantification of Goblet-like cells for wt (*n* = 3) and dko (*n* = 3) mice is shown (at least 10 crypts per mouse). Significance (**b**, **d** and **g**, **h**) was determined using a two-sided *t*-test and is expressed as the mean ± SEM. **i** Volcano plot showing the differentially (fold-change >1.5 and FDR <0.05) up- and down- regulated genes of PC/GC cluster. **j** UMAP and violin plots showing the expression of Paneth signature genes in wt and dko samples. **k** UMAP and violin plots showing the expression of Goblet signature genes in wt and dko samples. In UMAP gene expression projections, red indicates maximum gene expression while blue indicates low or no expression in log-normalized UMI counts. In violin plots, the X axes depict intestinal clusters subpopulations and the Y axes represent gene expression in log-normalized UMI counts. Source data are provided as a Source Data file.

Our scRNA-seq analysis indicated that not only the mature secretory cells were affected in dko SI but also cluster 8 cells (Fig. 3b). Cluster 8 cells expressed genes associated with common progenitors of Paneth and Goblet cells including *Spink4*^30^ (Supp. Fig. 6b). Based on these gene expression patterns and our trajectory analysis, we named this population Paneth/Goblet progenitors (PC/GC). Consistent with a Paneth cell differentiation block, the proportion of these cells was notably increased in the dko compared to wt mice (Fig. 3b). Proliferation characterizes immature intestinal cells, thus, to validate the increase in progenitors we stained intestinal sections with anti-Ki-67, a proliferation marker. As expected, post-mitotic Paneth cells were negative for Ki-67 staining in wt sections. However, all cells in the crypts stained positively for Ki-67 in dko sections (Fig. 4a, b), indicating that all crypt cells are dividing stem cells, progenitors, and immature IECs. These results were supported by cell cycle analysis of our scRNA-seq dataset, which showed an increase in proliferation in SC, PC, and PC/GC cells in dko mice (Supp. Fig. 6c). Furthermore, we stained the intestinal sections for OLFM4, a stem and progenitor cells marker. Comparable to Ki-67 staining, all cells in the crypts stained positively for OLFM4 in dko mice (Fig. 4c, d). Together, these results show that the intestinal crypts of dko mice are enriched for immature proliferating cells.

To further validate that crypts of dko mice are enriched for PC/GC progenitors, we double-stained intestinal sections with anti-LYZ1 (Paneth cells) and Alcian Blue (Goblet cells). As expected, differentiated Paneth cells were only found at the bottom of wt crypts, whereas Goblet cells were located in the upper part (Fig. 4e). Nonetheless, in dko mice, cells stained with Alcian Blue were identified considerably closer to the crypt base, suggesting the existence of a Goblet-like cell type in this unusual location (Fig. 4e).

Then, we performed double staining using PAS and Alcian Blue to identify secretory progenitor cells^31^. While we found that the number of Goblet cells decreased in the dko jejunum villi as compared to wt villi, the number of PC/GC progenitors increased in the dko crypts (Fig. 4f, g). To strengthen these findings, we quantitated the number of AB/PAS-positively stained PC/GC in the inducible dko mice showing that the number of progenitor cells increased in these mice, as well (Supp. Fig. 6d). Lastly, we carried out single-molecule fluorescence in situ hybridization (smFISH) using two RNA probes: Matrix Metalloproteinase 7 (*Mmp7*; Paneth) and Mucin 2 (*Muc2*; Goblet). Of note, the combined expression of these two genes was found enriched in the PC/GC progenitor cluster (Supp. Fig. 6e). We focused our analysis on double-expressing cells present at the crypt base. Indeed, the dko crypts were highly enriched for cells expressing Paneth and Goblet markers, as compared to wt crypts (Fig. 4h). Collectively, the immunohistochemical, scRNA-seq, and smFISH analyses clearly show that in the absence of TET2/3, the PC/GC progenitors become more abundant at the crypt base.

To further characterize these PC/GC progenitors at the transcriptomic level, we identified the differentially expressed genes in dko mice in our scRNA-seq dataset. Interestingly, most of the downregulated genes were effector Paneth-specific genes (Fig. 4i), yet again highlighting the blockage of Paneth cell differentiation upon TET2/3 depletion. In addition, we re-examined the expression patterns of Paneth and Goblet gene signatures across all clusters, observing an overall reduction in the Paneth signature in the dko samples (Fig. 4j). In contrast, we observed an increased expression level of the Goblet signature in the dko PC1 cluster compared to wt PC1, as well as an increase in Goblet-specific transcription factor KLF4^32^ (Fig. 4k and Supp. Fig. 6f), further supporting the staining experiments. Thus, suggesting that the remaining Paneth cells in dko mice crypts display a more immature or PC/GC-like phenotype and further supporting our immune-histological analyses. Taken together, our results revealed that Paneth/Goblet progenitor cells are expanded in dko mice, and PC/GC together with PC cells remain in an immature state in dko mice, both in the constitutive and the inducible dko.

### TET2/3 loss induces profound epigenetic changes at regulatory elements

To analyze the impact of TET2/3 dko on gene transcription in more depth, we performed bulk RNA-seq of IECs that were isolated from the jejunum part of wt and dko mice (Supp. Table 2). Principal component analysis indicated substantial similarity among the three wt replicates as opposed to the three dko samples (Supp. Fig. 7a). Differential gene expression analysis identified 400 genes that were significantly (*q* ≤ 0.05 and ≥2-fold-change) upregulated and 557 genes that were downregulated in dko samples in comparison to their wt counterparts (Supp. Fig. 7b), indicating substantial TET2/3-controlled gene expression. GO analysis of the upregulated genes revealed an enrichment of genes involved in immune and defense responses (Supp. Fig. 7c), whereas pathway analysis of the 557 downregulated genes showed enrichment in metabolic and oxidation-reduction processes (Supp. Fig. 7d). Gene Set Enrichment Analysis (GSEA) also indicated that the Paneth cells gene set is considerably decreased in the dko mice (Supp. Fig. 7e). The results also indicated that type II Tuft cells signature was decreased in dko while type I Tuft cells signature appeared unaffected (Supp. Fig. 7f, g). Altogether, our single-cell and bulk RNA-seq, and immune-histochemical staining results demonstrate highly consistent effects of TET2/3 on gut epithelial cell biology.

To determine the genome-wide impact of TET2/3 dko on DNA methylation, we performed whole-genome bisulfite sequencing on jejunum crypt IECs. Three biological replicates from each genotype were sequenced (Supp. Table 3). Data analysis revealed that global methylation levels were robustly increased in the dko samples (Supp. Fig. 8a), consistent with the loss of TET-dependent DNA demethylation. In the absence of TET2/3, numerous (*N* = 5830) promoters gained DNA methylation while few (*N* = 163) promoters lost methylation (≥10% methylation change, Supp. Fig. 8b). We then focused on the hypermethylated promoters since we considered them as a potential direct consequence of TET2/3 loss, and intersected gene expression datasets with the hypermethylated promoters. This analysis identified 472 promoters linked to gene expression changes (Supp. Fig. 8c). Using published ChIP-seq data obtained from wt and SI IECs, we showed that these 472 hypermethylated promoters are enriched for H3K4me3 and H3K27ac, thus, suggesting that they are actively transcribed under normal conditions (Supp. Fig. 8e). Consistently, these hypermethylated promoters were linked to 192 genes with downregulated expression (FC ≤ 2, *q* value ≤ 0.05) and to 96 genes with upregulated expression (FC ≥ 2, *q* value ≤ 0.05) (Supp. Fig. 8d). Interestingly, the 192 genes that were downregulated in *Tet2/3* dko mice were substantially repressed in colitis (Supp. Fig. 8f).

**Fig. 5:**
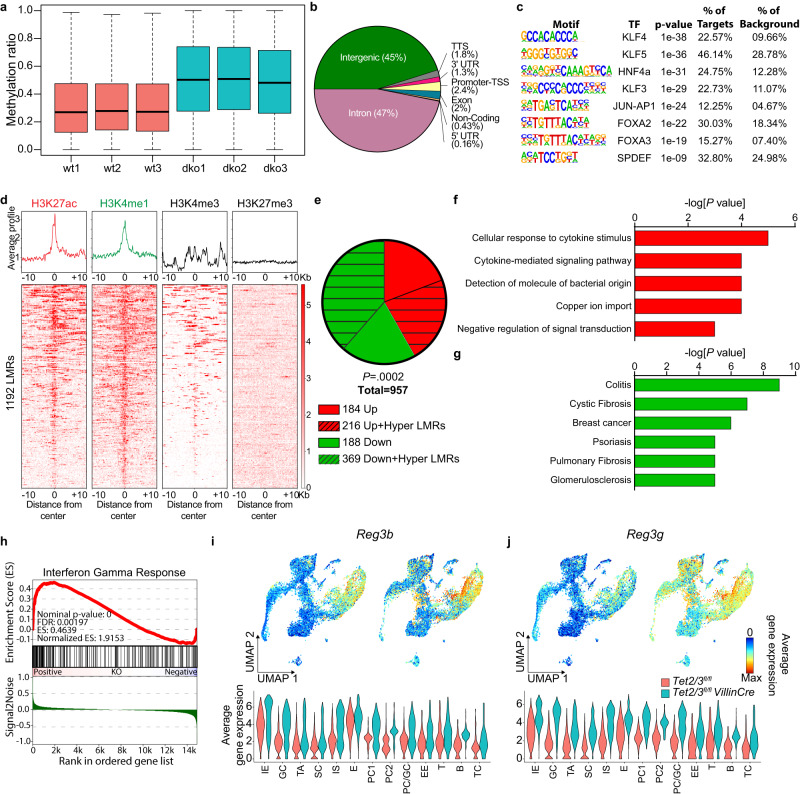
Loss of TET2/3 induces profound changes at enhancer regions. **a** Boxplot of all LMRs (*n* = 210,045) identified in wt (*n* = 3) and dko (*n* = 3) mice. Boxplot shows the median, the 25th and 75th percentiles, and the smallest and largest values within 1.5× the interquartile range (whiskers). **b** Pie chart showing the proportion of LMRs that overlap each feature. **c** Motif analysis of the hypermethylated LMRs (*n* = 1192) that are associated with significantly reduced gene expression in dko, motifs with the highest scores are listed. *P* values were calculated using the cumulative hypergeometric distribution (a one-tailed test). **d** Average histone modification profiles of hypermethylated LMRs. The normalized signal of different histone modifications was measured in a window of ±10,000 base-pair (bp). **e** Pie chart showing the number of significantly upregulated (red slice) and downregulated (green slice) genes. The highlighted slices represent the proportion of the genes that were associated with hypermethylated LMR (*P* value was calculated using a two-tailed Fisher’s exact test). **f** GO analysis of the 216 upregulated genes from (**e**). The highly enriched biological processes from the enriched categories are shown. **g** Diseases associated with the 369 downregulated genes associated with hypermethylated LMRs in dko versus wt samples. *P* values (**f**, **g**) were calculated using a two-tailed Fisher’s exact test. **h** GSEA analysis based on bulk RNA-seq data for intestinal cells from wt and dko mice. Enrichment is shown for interferon-gamma response signature increased in dko mice. Statistics were generated in accordance with the published GSEA algorithm. **i**, **j** UMAP and violin plots showing the expression of **i** *Reg3b* and **j** *Reg3g* in wt and dko samples. In UMAP gene expression projections, red indicates maximum gene expression while blue indicates low or no expression in log-normalized UMI counts. In violin plots, X-axis depicts intestinal cluster subpopulations and Y-axis represents gene expression in log-normalized UMI counts.

To further refine our DNA methylation analysis, we then focused on low-methylated regions (LMRs), which represent active regulatory regions such as enhancers. This analysis revealed pronounced hypermethylation of LMRs in the SI of dko mice (Fig. 5a). In total, we identified 46,877 LMRs that gained (methylation difference > 0.2) DNA methylation in the absence of TET2/3 (designated hypermethylated LMRs), whereas only 346 showed decreased methylation levels (designated hypomethylated LMRs). Subsequently, we determined the distribution of all 46,877 hypermethylated LMRs across the genome. We found that the majority of the hypermethylated LMRs are located within genic regions (Fig. 5b). Since LMRs provide an opportunity to overlap DNA methylation changes with gene expression changes, we assigned these LMRs to the nearest gene. This identified 1,192 hypermethylated LMRs that are associated with significantly repressed genes in dko SI IECs. Interestingly, these LMRs were significantly enriched for a variety of predicted transcription factor binding sites (Fig. 5c), including FOXA2,3, KLF3,4,5 (known to play an important role in gut development, morphology, and homeostasis and to recruit TET enzymes to chromatin), SPDEF, HNF4A, and AP1^33–36^. Furthermore, we examined these LMRs for chromatin marks, using published ChIP-seq data. Our results show that these hypermethylated LMRs were distinctly enriched for the enhancer chromatin marks H3K4me1 and H3K27ac, and depleted for the active promoter mark H3K4me3 and the repressive mark H3K27me3 in wt IECs (Fig. 5d). Together, these results suggest that deletion of TET2/3 induces profound epigenetic changes at putative enhancer regions in the SI.

Of note, 622 hypermethylated LMRs were associated with 216 genes that showed an increased expression level, and 1192 hypermethylated LMRs were associated with 369 downregulated genes (FC ≥2, *q* ≤ 0.05) in TET2/3 dko mice (*p* = 0.0002, Fisher’s exact test) (Fig. 5e). GO analysis of the 216 upregulated genes, showed enrichment for immune response pathways (Fig. 5f), and the 369 downregulated genes were enriched for colitis pathways (Fig. 5g and Supp. Fig. 8g). The GSEA analysis of the upregulated genes revealed the activation of the interferon-gamma response pathway, a known inflammatory-specific pathway (Fig. 5h). Moreover, the expression of *Reg3b* and *Reg3g*, which are upregulated in response to intestinal inflammation, was globally increased in all cell types of the dko in our scRNA-seq analysis, corroborating the bulk analysis (Fig. 5i, j). Importantly, about 30% of the genes in both groups showed association with only one LMR, whereas about 50% showed linkage to 2–4 LMRs per gene (Supp. Fig. 8h, i), thus further emphasizing the role that these elements play in the methylation-dependent regulation of SI homeostasis.

**Fig. 6:**
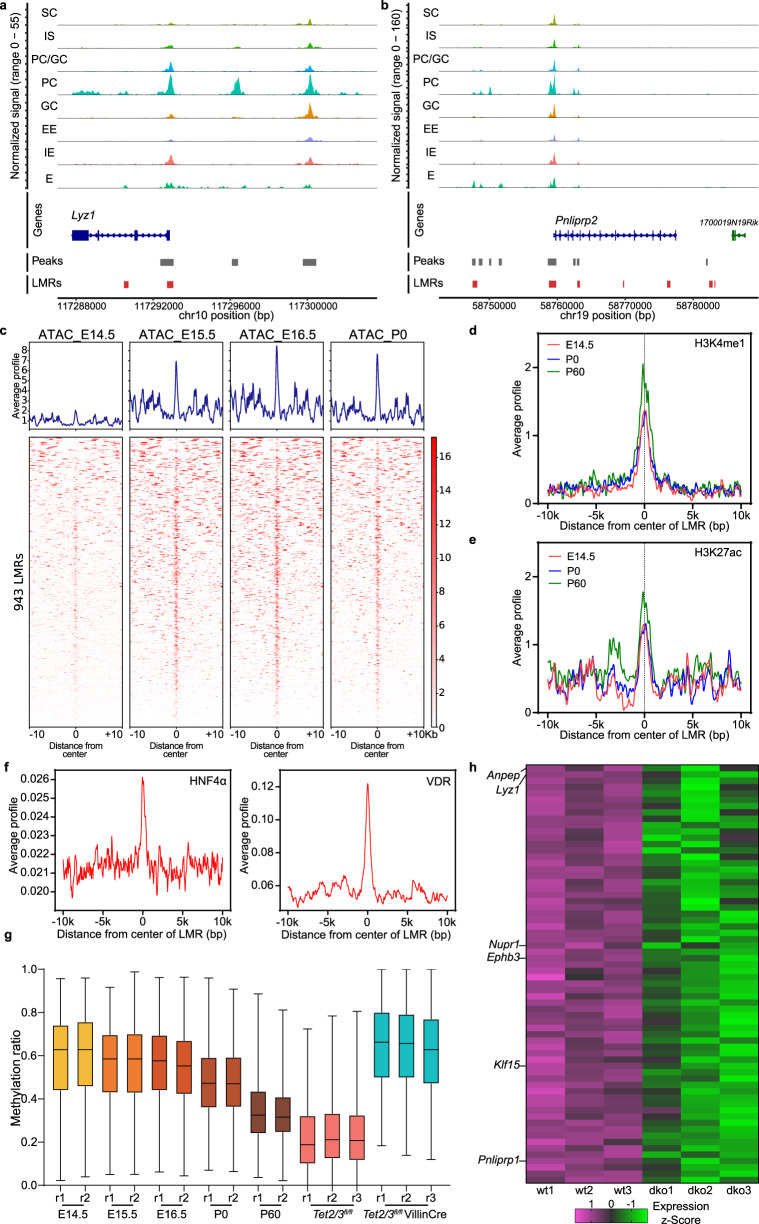
TET2/3 induces profound epigenetic changes at putative enhancer regions required for Paneth differentiation. **a**, **b** Displayed is a genomic region up- and down-stream of the PC-specific genes *Lyz1* (**a**) and *Pnliprp2* (**b**) showing scATAC profile of several intestinal cell types and hypermethylated LMRs. Gray-colored boxes represent scATAC peaks. Red colored boxes represent hypermethylated LMRs in dko mice. **c** Heatmap shows signals of published ATAC-seq data over the 943 hypermethylated LMRs that are associated with scATAC peaks of Paneth cells. **d** Average H3K4me1 modification profiles of 943 hypermethylated LMRs. The normalized signal of different histone modifications measured in a window of ±10,000 bp. **e** Average H3K27ac modification profiles of 943 hypermethylated LMRs. The normalized signal of different histone modifications was measured in a window of ±10,000 bp. **f** Average profile of different transcription factors (HNF4A and VDR) over the 943 hypermethylated LMRs. The normalized signal of different transcription factors was measured in a window of ±10,000 bp. **g** Boxplot showing the average methylation level of 943 hypermethylated LMRs that are associated with Paneth-specific genes during the indicated developmental time points. Two replicates (r1 and r2) are shown for each analyzed sample. Boxplot shows the median, the 25th and 75th percentiles, and the smallest and largest values within 1.5× the interquartile range (whiskers). **h** Heatmap of 66 downregulated genes that are associated with 943 hypermethylated LMRs.

Given the block in Paneth cell differentiation observed in the scRNA-seq analysis, we set out to identify Paneth-specific candidate genes that are potential direct targets of TET2/3. To address this question, we searched available chromatin accessibility (ATAC-seq) datasets for scATAC Paneth-specific peaks^37^ that overlap with hyper-methylated LMRs identified in dko mice. Out of the 1795 Paneth-specific ATAC-seq peaks we identified 943 that were associated with LMRs. As illustrative examples, we analyzed the chromatin accessibility at two Paneth-specific genes (*Lyz1* and *Pnliprp2*) that were associated with hypermethylated LMRs and which showed the expected dynamics by showing the highest accessibility in Paneth cells (Fig. 6a, b). Interestingly, we also observed higher co-accessibility between ATAC and LMR regions and additional nearby peaks uniquely or specifically in Paneth cells, thus, suggesting a *cis*-regulatory relationship between them (Supp. Fig. 9a, b).

These chromatin-accessible-LMR regions start to appear at E14.5 and become more open in subsequent embryonic days. They are also marked with H3K4me1 and to a lesser degree with H3K27ac. Both histone modifications are more pronounced in the adult intestine (i.e., P60), suggesting that they are environmental-dependent (Fig. 6d, e). Interestingly, published ChIP-seq data clearly show that the transcription factors HNF4a, VDR, and PRDM16 are bound to these accessible-LMR regions (Fig. 6f and Supp. Fig. 9c), altogether suggesting that these elements are putative enhancers. These transcription factors are involved in Paneth cell development and differentiation^38–41^, and can bind to and recruit TET2 and TET3^42,43^.

Strikingly, published DNA methylation data shows that these accessible-LMR regions are methylated during earlier stages of development and undergo DNA demethylation in the adult intestine (Fig. 6g). Since the ATAC data was obtained during embryogenesis, our data strongly suggest that DNA demethylation of these regulatory regions, occurs after chromatin opening. Of note, the DNA methylation level of the accessible-LMR dko elements is comparable to the E14.5 pattern (Fig. 6g), indicating that TET2/3 enzymatic activity occurs postnatally. Finally, the 943 accessible-LMRs regions were associated with 67 downregulated Paneth-specific genes (FC ≥ 1.5, *q* ≤ 0.1) in TET2/3 dko mice, including *Lyz1*, *Ephb3*, *Klf15*, etc., (Fig. 6h and Supp. Fig. 9d). In addition, the Matrix metalloproteinase 7 (MMP7), which regulates the activation of the antimicrobial α-defensins that are generated by Paneth cells^44^, was also repressed and showed a hypermethylated promoter in dko mice (Supp. Fig. 9e). These results support the notion that Paneth cells from dko crypts are immature, and therefore unable to protect the intestine from inflammatory signals.

We also evaluated whether there is a similar effect on Tuft cell differentiation, another cell population that we had found to be reduced upon TET2/3 loss. Thus, we searched our bulk RNA-seq data for Tuft cell-specific genes^28^ that are downregulated in dko as compared to wt mice, and linked to hypermethylated LMRs (Supp. Fig. 10a). Also, these LMRs are hypermethylated during embryonal development and undergo demethylation in the adult (Supp. Fig. 10b). We were particularly interested in the expression of transcription factor genes that were differentially expressed between wt and dko cell populations, which may explain the reduction in Tuft cell number in dko mice. Indeed, this approach identified the Tuft master transcription factor gene POU2F3^45^, which is required for Tuft cell development^45^. More specifically, our data show that the *Pou2f3* gene is linked to two hypermethylated LMRs and is repressed more than twofold in dko crypts (Supp. Fig. 10c, d).

### DNA methylation inhibition partially rescues TET2/3 dko phenotype

**Fig. 7:**
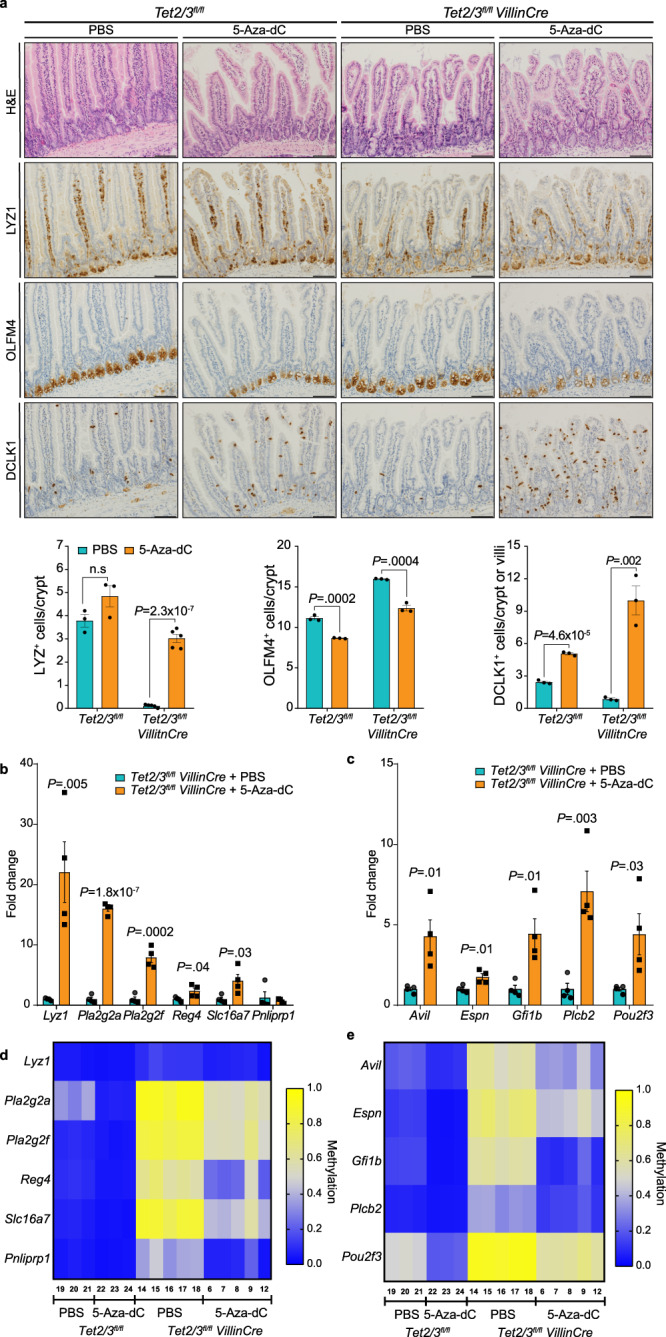
5-Aza-dC treatment partially rescues the loss of Paneth and Tuft cells. **a** H&E, LYZ1-, OLFM4-, and DCLK1- stained histologic images of the jejunum from wt and dko mice, treated with or without 5-Aza-dC. Quantification of LYZ1-, OLFM4- and DCLK1-positive cells are shown (*n* = 3, except for dko LYZ- positive cell where *n* = 5). Scale bar 100 µm. **b** Expression levels of several Paneth cell markers in untreated dko (*n* = 4) and 5-Aza-dC-treated (*n* = 4) mice. **c** Expression levels of several Tuft markers in untreated dko (*n* = 4) and 5-Aza-dC-treated dko (*n* = 4) mice. Significance (**a**–**c**) was determined using a two-sided *t*-test and is expressed as the mean ± SEM. **d** Bisulfite sequencing results for Paneth-specific LMRs defined in wt versus dko mice. The heatmap shows the average methylation ratios of five LMR amplicons from wt (*n* = 3) and dko- (*n* = 5) treated with or without 5-Aza-dC. The *Lyz1* gene is highly activated in 5-Aza-dC treatment, whereas its associated LMR shows very little methylation change. This could be explained by the fact that additional unidentified methylation-sensitive regulatory elements are likely involved in its regulation. **e** Bisulfite sequencing results for Tuft-specific LMRs defined in wt versus dko mice. The heatmap shows average methylation ratios of 5 LMR amplicons from wt (*n* = 3) and dko- (*n* = 5) treated with or without 5-Aza-dC. Source data are provided as a Source Data file.

Since the loss of TET2/3 causes global DNA hypermethylation and disrupts intestinal homeostasis, we attempted to rescue the dko phenotype by pharmacological inhibition of DNA methylation. To this end, we treated wt and dko mice with 5-Aza-2’-deoxycytidine (5-Aza-dC) at a very low dose (0.2 mg/kg) and found that this treatment had no effect on intestinal morphology in wt mice as determined by H&E staining (Fig. 7a). Remarkably, however, this treatment enabled immature Paneth cells to complete their differentiation to LYZ1-positive cells and to be positioned at the base of the crypts in dko mice (Fig. 7a). Moreover, OLFM4 staining revealed a decrease in the number of stem and progenitor cells in the 5-Aza-dC-treated dko mice compared with wt mice (Fig. 7a). Interestingly, DCLK1 staining indicated a significant increase in the Tuft cell number in the 5-Aza-dC-treated dko mice (Fig. 7a).

Finally, we observed an increased expression of several Paneth and Tuft cell-specific gene markers in the 5-Aza-dC-treated TET2/3 dko, compared to untreated dko mice (Fig. 7b, c). Furthermore, targeted bisulfite analysis confirmed that 5-Aza-dC treatment induced DNA demethylation at several LMRs that were associated with these markers (Fig. 7d, e). Interestingly, 5-Aza-dC treatment could rescue the DNA methylation and expression levels of the developmental transcription factor POU2F3, explaining the upregulation of Tuft cells in the intestine of treated dko mice (Fig. 7a). Collectively, our data clearly shows that TET2/3 are critical for expression of a master transcription factor which leads to normal Tuft cell development. Furthermore, these findings clearly support the notion that TET2/3 regulate intestinal differentiation and homeostasis through their catalytic activities.

### Microbiome profiling reveals altered gut microbiota in TET2/3 dko mice

**Fig. 8:**
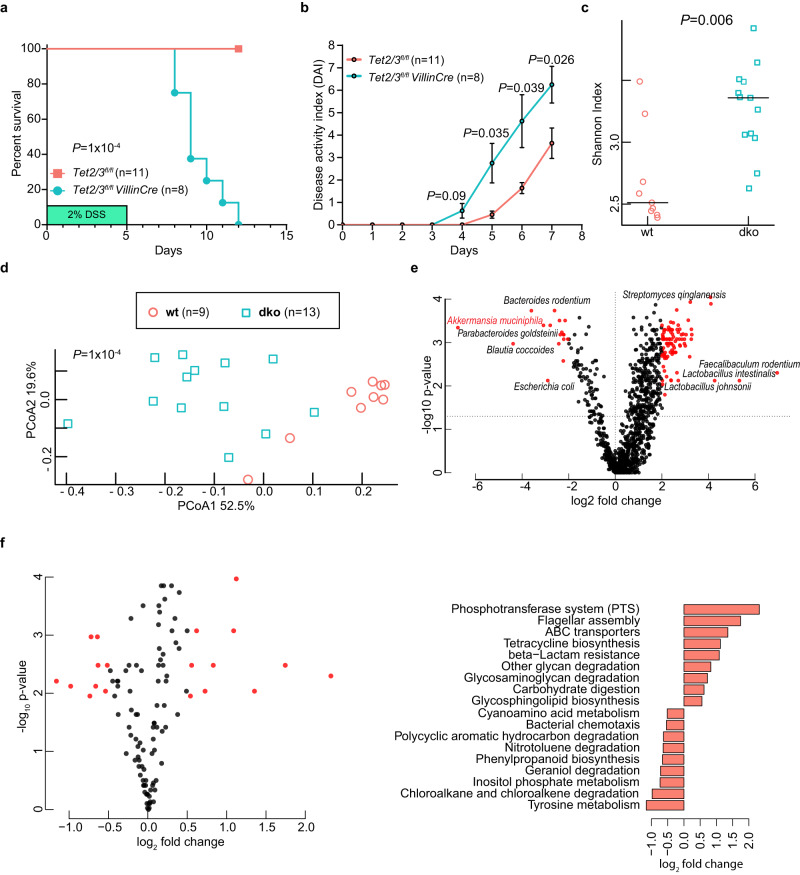
Altered gut microbiota in TET2/3 dko mice. **a** Survival curve for 8-week-old *Tet2/3*^*fl/fl*^ (*n* = 11) and *Tet2/3*^*fl/fl*^ *VillinCre* (*n* = 8) littermates treated with 2% DSS for 5 days. The mice were then followed until day 12. **b** Disease activity index (DAI, see Supp. Table 4) was monitored daily for both *Tet2/3*^*fl/fl*^ (*n* = 11) and *Tet2/3*^*fl/fl*^ *VillinCre* (*n* = 8) mice. Significance (**a**, **b**) was determined using a two-sided *t*-test and is expressed as the mean ± SEM. **c** Alpha diversity measured by Shannon diversity index in wt (*n* = 9) and dko (*n* = 13) mice. The larger Shannon index value indicates higher community diversity. Significance was determined using a two-sided Wilcoxon test and is expressed as the mean ± SEM. **d** PCA plots of beta diversity analysis of wt (*n* = 9) and dko (*n* = 13) mice. *P* value was calculated using the PERMANOVA test. **e** Volcano plot showing the significantly enriched (right) and depleted (left) bacteria in dko (*n* = 13) compared with wt (*n* = 9), using DESeq2 with Benjamini–Hochberg correction. **f** Analysis of the dko microbiota’s functional metabolic potential. Functional pathways were inferred from gene clusters and the differential abundance testing was performed using two-sided Wilcoxon tests with FDR correction (features with negative values enriched in wt). Source data are provided as a Source Data file.

Our scRNA-seq as well as our combined analysis of bulk transcriptomics and DNA methylation clearly suggest that the dko SI is primed for inflammation. Thus, we next asked what is the functional consequence of this phenotype. To that end, we examined the sensitivity of wt and dko mice to Dextran Sulfate Sodium (DSS) treatment, a colitis-inducing compound. Strikingly, after 7 days of treatment, all wt mice survived the treatment whereas all dko mice started dying, and by day 12 the dko had all died (Fig. 8a). Moreover, we analysed the disease activity score of wt and dko mice during the first 7 days of the experiments (prior to death) (Fig. 8b). Indeed, the disease activity index (DAI, see Supp. Table 4) of dko mice is significantly higher than the wt mice. Altogether, our results show that the functional consequences of aberrant DNA methylation of thousands of regulatory elements, that are required for proper cellular homeostasis, results in excessive response to inflammatory signals.

Our analyses show a reduction of Paneth and Tuft cells in the intestinal crypts of dko mice, which have been shown to regulate the composition of the intestinal microbiota. Thus, we hypothesized that their reduction could result in an altered microbiome in dko mice. To test this hypothesis, we collected feces (Fig. 8c–f), as well as the content of the small intestine (Supp. Fig. 11), from wt and dko mice and analyzed the composition of the intestinal microbiota by metagenomic shotgun sequencing. Initially, we analyzed the diversity of the microbiota within each sample (alpha diversity). Interestingly, we found that the dko mice exhibit a significantly higher Shannon index compared to wt mice (*p* = 0.006, Wilcoxon test) in the feces (Fig. 8c), indicating a higher average community diversity in the dko fecal samples. Most of the wt samples (both fecal and SI) clustered together while the dko samples were more scattered and clustered distinctly from the wt samples (Fig. 8d and Supp. Fig. 11b). These results indicate a high degree of similarity among wt samples, while the dko samples are both more heterogeneous and dissimilar from the wt samples (*p* = 0.0001, Wilcoxon test).

Furthermore, we analyzed the significantly enriched and depleted microorganisms in the feces of dko samples. The results showed a highly significant reduction for *Akkermansia muciniphila* (Fig. 8e). Recently, it has been shown that *Akkermansia muciniphila* species can secrete a glucagon-like peptide-1 (GLP1)-inducing protein that enhances glucose homeostasis and ameliorates metabolic disease in mice^46^. Furthermore, the dko samples are characterized by a reduced metabolic capacity to metabolize tyrosine, inositol phosphate, and cyanoaminoacid, as well as to degrade chloroalkane and nitrotoluene. On the other hand, bacterial phosphotransferase system encoding genes and flagellar assembly are increased in the microbiota of dko mice (Fig. 8f).

We have performed a similar analysis on the significantly enriched and depleted microorganisms in the SI dko samples. The results showed a highly significant reduction for *Porphyromonadaceae bacterium UBA3321*, and an increase in *Candidatus Arthromitus* (SFB), and *Chlamydia muridarum* (Supp. Fig. 11c), known to modulate or alter adaptive host immune response in mice^47^. In summary, these findings are consistent with the notion that the loss of TET2/3 results in excessive response to inflammatory signals, and in altered microbiome composition (Fig. 8 and Supp. Fig.11), which possibly instructs an intestinal pro-inflammatory phenotype (Fig. 5 and Supp. Fig. 7).

## Discussion

The role of TET proteins in cellular differentiation and tissue homeostasis is much less well understood and largely limited to the hematopoietic system and the brain^13,14,48,49^. Our results show that loss of TET2/3 in vivo disrupted differentiation and homeostasis of the SI, thus illustrating how intestinal differentiation is sensitive to changes in DNA methylation^21^. More specifically, we observed ablation of mature Paneth cells, reduced numbers of Tuft cells, aberrant location of Goblet-like cells, and increased numbers of EE cells. This phenotype was observed upon deletion at day E14.5, as well as upon deletion at P1, indicating that TET2/3 demethylating activity is required postnatally. Since the most noticeable change is the loss of Paneth cells, this may suggest that DNA methylation changes influence Paneth cell fate decision more than other intestinal cell types, similar to the influence DNA methylation exerts on myeloid *versus* lymphoid cell fate decision^50,51^.

The loss of Paneth cells was sex-independent and required ablating both *Tet2* and *Tet3*, suggesting redundant functions of the two enzymes in the SI. Although these enzymes might also exhibit other activities^52–54^, the effects of 5-Aza-dC induced DNA demethylation, which resulted in the rescue of Paneth and Tuft cells, thus, clearly supported the notion that the catalytic activity of TET2/3 enzymes regulates intestinal differentiation and homeostasis. Furthermore, it highlights the antagonistic activities of DNA-methylating and DNA-demethylating enzymes within the intestinal epithelium, as previously shown in embryonic and hematopoietic stem cells^12,55,56^. This suggestion is also supported by previously published data showing that although cell fate decisions among differentiating cells were largely unaffected by the loss of *Dnmt1*, increased numbers of secretory progenitor cells were found in the mouse intestine, suggesting a partial disruption in secretory cell specification^21^.

The *Tet2/3* dko mice displayed a very different phenotype to the one observed in the intestinal *Tet1* ko mice^57^, which was growth-retarded, and exhibited partial postnatal lethality. *Tet1* is expressed at much higher levels in stem cells than in differentiated cells, while *Tet2* and *Tet3* show the opposite pattern^23,57^. Correspondingly, TET1 might have an essential role in stem cell function, whereas TET2/3 are responsible for gene regulation in more differentiated cells. It also may explain our data showing that the first noticeable change in intestinal homeostasis is detected in progenitor cells rather than in stem cells.

We identified a vast number of gene regulatory elements which were hypermethylated in the dko mice and were associated with hundreds of genes connected to normal and pathological disorders in the SI, thereby providing additional evidence for a functional role of active DNA demethylation in proper intestinal cell fate specification and tissue homeostasis. Of note, the hypermethylated LMRs were associated with changes in gene expression and were enriched for enhancer marks (H3K27ac and H3K4me1), suggesting that this set of LMRs are putative enhancers. The hypermethylated LMRs were also enriched for a variety of transcription factor binding sites. These transcription factors are known to play a crucial role in intestinal homeostasis. For example, we observed the enrichment of KLF transcription factor binding sites within these LMRs. Recently, it has been reported that KLF4 interacts directly with TET2 and recruits it to specific DNA sites, prior to chromatin opening^58^. Moreover, it has been reported that KLF5 is needed for proper Paneth cell differentiation in the intestine^59^. Furthermore, SPDEF, HNF4A, and FOXA binding sites were also enriched in the hypermethylated LMR set. These transcription factors are all known to play an important role in intestinal differentiation and homeostasis^60–62^. It is, therefore, possible that the binding of many transcription factors to these LMRs is inhibited by DNA methylation, which may lead to changes in cellular differentiation and homeostasis.

Our findings also provide mechanistic explanations for the disrupted SI differentiation and homeostasis. It appears that the loss of Paneth cells, arises from direct and indirect mechanisms. For example, the loss of TET2/3 generates a developmental block resulting in immature Paneth and PC/GC progenitor cells. Furthermore, GO analysis of the downregulated genes in the remaining dko Paneth cells showed a reduction in genes associated with antimicrobial response. These antimicrobial genes are exclusively produced in mature Paneth cells^29^, indicating that the cells are not fully differentiated. Remarkably, high expression of ribosomal genes was observed in Paneth cells in the absence of TET2/3, correlating with the ability of progenitor cells to synthesize higher levels of ribosomal transcripts compared to their terminally differentiated counterparts^63^. Moreover, it has been shown that high expression of ribosomal genes delays cellular differentiation, whereas its downregulation induces differentiation^64–66^. In addition, Paneth cells also acquired a more Goblet-like expression pattern in dko mice, thus resembling PC/GC progenitors. Taken together, our findings suggest that, in the absence of TET2/3, Paneth cell differentiation is blocked at an immature stage, which can support the ISC niche but compromises their ability to eliminate pathogens.

Moreover, our results showed that the dko mice also harbor a general increase in EE, a decreased proportion of EE progenitors and an increased proportion of X (Ghrl) and L (Gcg) mature EE cells. The increased number of EE cells is associated with increased expression levels of EE transcription factors; *Neurod1*, *Neurog3*, *Nkx2-2*, and *Pax6* in dko mice. Interestingly, intestinal *Neurod1* expression impairs Paneth cell differentiation and promotes EE lineage specification^67^, consistent with the *Tet2/3* dko phenotype. We suggest that in the absence of TET2/3, regulatory elements that are methylated are unable to bind transcriptional repressors, or they favor binding of transcription factors that prefer methylated target sequences^68^, culminating in higher expression levels of their associated cell-specific transcription factors, resulting in higher numbers of mature EE cells.

To study whether TE2/3 directly affects Paneth-specific genes we integrated scATAC Paneth-specific peaks^37^ with the hypermethylated LMRs and expression changes identified in dko mice. We identified 67 downregulated Paneth-specific genes in TET2/3 dko mice, including *Lyz1*, *Ephb3*, *Klf15*, etc., most probably representing direct targets of TET2/3. In addition, it was previously shown that the proteolytic activity of MMP7 is required for Paneth cells to activate the precursor of alpha-defensins^69^. In our analysis, the *Mmp7* promoter is hypermethylated and its expression is reduced, resulting in a reduced expression of defensins, indicating yet again that DNA methylation is needed for the proper function of Paneth cells. Altogether, this data validates the regulatory role of these putative Paneth-specific regulatory regions and further links these TET2/3-induced changes to the impaired Paneth cell differentiation. Interestingly, our analysis clearly shows that these LMRs are located in accessible chromatin regions during embryonic development and undergo demethylation only postnatally. These novel findings strongly suggest that chromatin opening occurs prior to DNA demethylation, possibly via transcription factor binding.

Tuft cells indirectly regulate the gut microbiome through type two immune response. Notably, they trigger innate lymphoid cell type 2 (ILC2s) to secrete IL-13 to drive rapid response against bacteria and helminths^70,71^. The molecular mechanisms underlying the decrease in Tuft cells are similar to those found for Paneth cells; i.e., hypermethylation of putative enhancers which regulate tissue-specific expression of cell fate-determining transcription factors, such as *Pou2f3* and *Gfi1b*, as well as Tuft effectors, such as *Alox5ap*.

A direct outcome of the loss of Paneth and Tuft cells is the microbiome alteration observed in the dko SI and feces. Interestingly, the SI metagenomic analysis showed a highly significant reduction for *Porphyromonadaceae* bacterium UBA3321, and an increase in *Candidatus arthromitus* (SFB), and *Chlamydia muridarum*, which are known to alter adaptive host immune response in mice^47^. Moreover, in the feces, the most strongly depleted species in dko, *Akkermansia muciniphila*, is widely regarded to promote intestinal homeostasis. Thus, a decline in *A. muciniphila* may render mice more prone to intestinal inflammation^72^. Indeed, our current results clearly shows that TET2/3-deletion causes unrestrained inflammatory response resulting in early death. In addition, a potential link exists between the alterations observed on the microbiome functional level and the increased sensitivity to intestinal inflammation in dko mice. For example, the phosphotransferase system pathway (which is upregulated in the dko microbiome) is known to be linked to oxidative stress and intestinal inflammation in humans and mice^73,74^. In addition, the flagella products, which are also increased in dko mice are recognized by enteric TLR5 receptors^75^ and may thus also contribute to the pro-inflammatory gut microenvironment, as well.

In summary, our study clearly shows that TET2/3 integrates intrinsic and extrinsic stimuli to regulate intestinal homeostasis and cellular differentiation through DNA demethylation. In their absence, a block in secretory cell differentiation occurs. Moreover, TET2/3 loss results in the inability of the gut to properly accommodate the microbiome, resulting in an altered microbiome composition which contributes to a pro-inflammatory intestinal phenotype under homeostatic conditions, and acute inflammation-induced death.

## Methods

### *Tet2/3* knockout mice

All animal procedures were approved by The Animal Care and Use Committee of The Hebrew University of Jerusalem (MD-22-17012-5). Mice were housed and cared for under SPF conditions. Mice were maintained on a 12 h light and dark cycle, at 22 ± 2 °C, and 55 ± 15% humidity. All experiments used male mice, unless otherwise stated in the figure legend.

*Tet2/3*^*fl/fl*^ mice have been previously described in refs. ^13,23^. All mice are C57BL/6 or have been backcrossed to the C57BL/6 background. We crossed *Tet2/3*^*fl/fl*^ with *VillinCre* mice^76^ to generate *Tet2/3*^*fl/fl*^ and *Tet2/3*^*fl/fl*^ *VillinCre* mice. Adult mice of these strains were used between 8 and 10 weeks of age. We treated mice with 5-Aza-dC at a very low dose (0.2 mg/kg) to inhibit DNA methylation. Briefly, to inhibit DNA methylation, we treated mice with 5-Aza-dC for 2 weeks (3 i.p injections/week) and then allowed them to recover for one more week. To determine whether the requirement of TET2/3 are needed during embryonic development or postnatally, we generated TET2/3 inducible mutant mice. For this, we crossed *Tet2/3*^*fl/fl*^ with *VillinCreER* mice^25^. To induce TET2/3 dko, pups at the age of postnatal day 1 (P1) were intra-gastrically-injected with Tamoxifen (1 mg/ml). At the age of P28, the mice were sacrificed and samples were collected (crypts and tissues).

For inflammation induction, mice (*Tet2/3*^*fl/fl*^ and *Tet2/3*^*fl/fl*^ *VillinCre*) were given 2% of 36–50 kDa DSS for 5 days in their drinking water. On day 6, mice received water and kept for an additional 7 days. Body weight, stool consistency and the presence of gross blood in feces were evaluated daily and scored for each mouse during the experimental period. The disease activity index (DAI) was calculated by the total score (body weight decrease + stool consistency + rectal bleeding) (Supp. Table 4).

### Histochemistry and immunohistochemistry staining

All tissues were taken for histological analysis by overnight fixation in 4% formaldehyde (Bio-Lab, Cat#: 6450305), followed by dehydration in 80% ethanol. Paraffin embedding was performed according to the standard protocols in the Pathology Unit, Hadassah Hospital, Jerusalem. For both histochemistry and immunohistochemistry of tissues, sections of 5 µm were prepared. Hematoxylin/Eosin (H&E) and periodic acid-Schiff stain (PAS) staining was performed according to the standard protocol^77^. For Alcian Blue (AB) staining, sections were deparaffinized in xylene and graded alcohols and brought to distilled water. Sections were incubated in AB solution (Abcam, ab150662) for 30 min, followed by a wash in running tap water for 2 min and then counterstained with Nuclear Fast Red (Biocare Medical, 05-M07006) for 5 min. For immunohistochemistry, sections were deparaffinized in xylene and graded alcohols, followed by antigen retrieval. Endogenous peroxidase was quenched by H_2_O_2_. Sections were then incubated overnight at 4 °C with primary antibodies: LYZ1 (1:1000, Abcam, ab-108508), DCLK1 (1:300, Cell Signaling, 62257), OLFM4 (1:400, Cell Signaling, 39141), CHGA (1:400, Abcam, ab-15160) and Ki-67 (1:200, Thermo Fisher Scientific, MA5-14520). ImmPRESS® HRP Horse Anti-Rabbit IgG Polymer Detection Kit, Peroxidase (Vector Laboratories, Cat# MP-7401, Lot#: ZF0906) was used as secondary antibodies, and sections were developed using ImmPACT® DAB Substrate Kit, Peroxidase (Vector Laboratories, Cat#: SK-4105, Lot#: ZF0830). Slides were counterstained with hematoxylin, dehydrated, and mounted with Entellan (Merck, Cat# 100869).

### Single-cell RNA-sequencing and analysis

Small intestine crypts were isolated from three wt and three *Tet2*/3 dko mice as previously described in ref. ^78^. Single-cell suspensions from intestinal crypts were obtained by enzymatic digestion, incubating crypts in MEM medium supplemented with 2 mg/ml Trypsin and 2000 U/ml DNAse I for 30 min at 37 °C in a water bath, as previously described^79^. Apoptotic and dead cells were removed from suspension using the Dead Cell Removal Kit (Miltenyi Biotec). Approximately 20,000 cells per sample were loaded into the Chromium Controller system, and libraries were subsequently prepared using the Chromium Single Cell 3’ Reagent Kit v.2 (10X Genomics), following the manufacturer’s instructions. Libraries were sequenced on a HiSeq 4000 device (Illumina) using paired-end (26 + 74 bp) settings for 100 cycles.

Cell Ranger v.2.1.0 (10X Genomics) was used for raw sequencing data processing followed by downstream analysis with Seurat (v.3.1.1)^80^. To remove low-quality cells and potential cell doublets, we filtered out cells expressing less than 200 genes or more than 4000 as well as cells with more than 5% reads mapping to mitochondrial genes. This quality control resulted in a final dataset consisting of 25,693 single-cell transcriptomes. To confirm the deletion of *Tet2/3*, we used the bulk RNA-seq data (See below, same mice were used for both bulk- and sc- RNA-seq) and calculated the average coverage by read per base for each exon of *Tet2* and *Tet3* (Supp. Table 5).

To correct for confounding batch effects in our data, we combined all *Tet2/3*^*fl/fl*^ and *Tet2/3*^*fl/fl*^ *VillinCre* samples using Seurat’s standard integration protocol^80^. We used 50 Canonical Correlation Analysis (CCA) dimensions throughout the integration protocol and a resolution of 0.4 for unsupervised clustering. Most representative genes for each cell cluster were identified using FindAllMarkers() function in the integrated dataset, while FindMarkers() was used to identify differentially expressed genes in a particular cluster between *Tet2/3*^*fl/fl*^ and *Tet2/3*^*fl/fl*^ *VillinCre* samples.

To identify the distinct epithelial cell types, we used the gene signatures defined by Haber et al. (2017)^28^. The average gene expression of the top ten genes expressed by each epithelial cell population was projected into UMAP and violin plots. For a more detailed analysis of the EE compartment, all EE cells were substracted and re-clustered using a resolution of 0.8. To identify functional EE subtypes, we used progenitor and hormone genes, as previously described in ref. ^27^.

Epithelial cell lineages and pseudotime inference were performed using the R package slingshot (v1.6.1)^81^ using the integrated scRNA-seq dataset excluding immune clusters. As an input, we used the first three UMAP components calculated by Seurat. Also, we selected cluster #3 (Stem cells) as a starting cluster and fully differentiated cell clusters #1 (Goblet cells), #5 (Enterocytes), #7 (Paneth cells), #9 (Enteroendocrine), and #12 (Tuft cells) as ending points for the inference.

Pseudotime-dependent gene expression was then analyzed using the R package TradeSeq (v1.5.06) by fitting a generalized additive model^82^. The gene expression of the 5,000 most variable genes in our dataset was modeled for each lineage in both conditions (*Tet2/3*^*fl/fl*^ and *Tet2/3*^*fl/fl*^ *VillinCre*). Genes differentially expressed along Paneth cell lineage between *Tet2/3*^*fl/fl*^ and *Tet2/3*^*fl/fl*^ *VillinCre* were identified using conditionTest() function.

### Single-cell ATAC-sequencing analysis

To analyze chromatin co-accessibility in LMR-associated regions of PC-specific genes we obtained the set of PC-specific scATAC peaks directly from the authors of a previous dataset of wt mice SI crypts^37^. Furthermore, to visualize illustrative examples of this analysis we re-analyzed this dataset, which consisted of Neurog3-traced tomato-positive cells after two daily doses of tamoxifen at 12, 48, and 72 h from Ngn3CreERT2;R26tdtomato;Lgr5-DTRGFP mice.

Raw sequencing data were obtained from a public database (GEO: GSE183299) and downstream analysis was performed using the Seurat (v.4.2.0)^80^ and Signac (v.1.8.0)^83^ packages. Low-quality cells were filtered out using Signac by removing those with less than 2000 or more than 100,000 counts in the scATAC-seq data, cells with less than 40% reads within peaks, as well as cells with a genome blacklist ratio <0.01. Furthermore, we also filtered out cells with nucleosome signal higher than 4 and a transcriptional start site (TSS) enrichment lower than 1. We then used MACS2 (v. 2.1.1.20160309) to call the ATAC peaks on each sample independently. To ensure comparability between samples, we created a common set of peaks by merging all intersecting peaks using the GenomicRanges package (v.1.48.0)^84^. We then merged and subsequently integrated the three scATAC-seq datasets using the standard protocol described in the Signac package. Thus, integration anchors were identified between the three merged scATAC-seq datasets and reciprocal LSI projection was calculated. Then the samples were integrated using the integration anchors and the LSI coordinates from the merged datasets using the IntegrateEmbeddings() function. Dimensionality reduction was then calculated into a UMAP using the first 50 integrated lsi dimensions. Unsupervised clustering of the integrated data was performed using 50 lsi dimensions and 1.2 resolution, which resulted in 20 cell clusters. Cell-type identity was established by analyzing the chromatin accessibility at the top gene markers identified in our scRNA-seq dataset in each of the defined cell-type populations.

Cis-regulatory interactions at the PC-specific genes *Lyz1* and *Pnliprp2* were predicted by identifying co-accessible peaks in each intestinal cell type independently using Cicero (v.1.3.8)^85^. Only Cicero connections with a co-accessibility score higher than 0.25 were plotted.

### Transcriptome sequencing analysis

mRNA was extracted from the intestinal crypt isolated from the small intestine. Transcriptome sequencing libraries were prepared using the TruSeq RNA Sample Preparation Kit (Illumina, San Diego, USA), according to the manufacturer’s instructions. Reads were trimmed to a maximal length of 80 bp and stretches of bases having a quality score <30 at the ends of the reads were removed. Reads were mapped using Tophat 2.0.6 (ref. ^86^). As a reference sequence for the transcriptome mapping we used the mm9 assembly of the mouse genome. The parameters used for mapping are: -N 5,–read-edit-dist 5, -g 1, -p 8, -r 20. All other parameters were kept at their default values. Differential expression was quantified using DESeq 1.10.1 (ref. ^87^) applying the built-in procedures for library normalization and estimation of variance and with Cuffdiff 2.0 (ref. ^88^). The resulting *P* values were subjected to multiple testing correction using built-in functions available in DESeq and Cuffdiff, respectively. Genes with a *Q* value smaller than 0.05 were considered differentially expressed. Gene ontology (GO) analysis and pathway analysis for gene sets were performed using EnrichR^89,90^.

### Shotgun metagenomics sequencing and processing

Murine stool DNA was extracted and purified using a Purelink Microbiome DNA purification kit (Invitrogen). Illumina libraries were prepared for shotgun sequencing using a Nextera DNA Sample Prep kit (Illumina, FC-121-1031), according to the manufacturer’s protocol, and sequenced on the Illumina NextSeq platform with a read length of 80 bp. Illumina’s bcl2fastq script was implemented to generate the fastq files. Reads were QC trimmed using Fastp (v0.20.1) and Trimmomatic choosing the parameters PE -threads 10 -phred33 -validatePairs ILLUMINACLIP:TruSeq3-PE.fa:2:30:10 LEADING:3 TRAILING:3 MINLEN:50(^91^). We used KneadData (v0.7.2) and Bowtie2 (v2.2.4) with default parameters to remove host reads using mm9 as the reference. The cleared fastq files were subsampled using Seqtk (v.1.2-r94-v.1.3-r114) (https://github.com/lh3/seqtk). We carried out the taxonomic assignment of bacterial DNA relying on the exact alignment of k-mers with Kraken2 (v2.1.2)^92^ against the Genome Taxonomy Database [https://gtdb.ecogenomic.org/]. Accuracy was improved using Bayesian re-estimation of bacterial abundances with Bracken v.2.6.2(^92^). We extracted the counts for species-level analysis and subsampled the count tables to the lowest sequencing depth on an experiment-wise basis (~500k for feces/350k for SI content). Additionally, we removed bacteria that failed to reach a total abundance of at least 0.01. Functional annotation was implemented using protein alignment with DIAMOND (v.2.0.15). Thereby only the first hit was considered. An e value <0.0001 was accepted. We then used EMPANADA [https://github.com/borenstein-lab/empanada] for sample-specific assignment of gene families to pathways. Numerical ecology analysis. We analyzed the microbial community ecology with R (v3.4.3-3.6.3), mainly relying on the vegan [https://rdrr.io/cran/vegan/man/vegan-package.html] and mixOmics [http://mixomics.org/] packages. For ordination, we performed principal component analysis applying center log-ratio transformation (CLR). Differential species abundance analysis was performed using the R package DESeq2 (v1.10.1-v1.12.3) [bioconductor.org/packages/release/bioc/html/DESeq2.html]. Differential abundance testing of individual bacterial species was carried out using Mann–Whitney *U*- test, and log2 fold changes were calculated. *P* values were adjusted using the FDR correction method.

### Whole-genome bisulfite sequencing and analysis

WGBS was performed on three biological replicates from *Tet2/3*^*fl/fl*^ and *Tet2/3*^*fl/fl*^ *VillinCre* mice. Library preparation for bisulfite sequencing was performed as described previously^93^. Reads were trimmed to a maximal length of 80 bp and stretches of bases having a quality score <30 at the ends of the reads were removed. Reads were mapped using BSMAP 2.5 (ref. ^94^). As a reference sequence for the bisulfite mapping we used the mm9 assembly of the mouse genome. Only reads mapping with both partners of the read pairs at the correct distance were used. The correct distance was defined by setting the minimum value to 50 bp and the maximum value to 800 bp. In case a read pair mapped to multiple sites on the reference sequence a random hit was chosen. This was done by setting the option -r of bsmap to 1. The maximum number of mismatches allowed was set to 4% of the number of bases of a read. The whole list of parameters used for mapping with BSMAP 2.5 are: -d mm9, -s 16, -v 0.04, -w 100, -r 1, -q 0, -z 33, -f 5, -A none, -B 1, -E 4,294,967,295, -L 144, -D none, -I 4, -S 0, -n 1, -M TC, -p 4, -m 50, -x 800. Duplicates were removed using the Picard tool [http://broadinstitute.github.io/picard]. Methylation ratios were determined using a Python script (methratio.py) distributed together with the BSMAP package. For both the forward and reverse strands, all cytosine bases in the GC context were called independently. LMRs were identified with MethylSeekR^95^.

For further analysis, we used chromHMM^96^, which performs Hidden Markov Modeling of input data. As training datasets we used available ChIP-seq tracks (http://genome.ucsc.edu) for H3K4me1, H3K4me3, H3K27ac, H3K27me3, and H3K36me3. This resulted in a segmentation of the genome into 15 different types of compartments, which were annotated based on the combination of related histone patterns. The motif analysis was performed using HOMER software^97^.

To compare the methylation levels of *Tet2/3*^*fl/fl*^ and *Tet2/3*^*fl/fl*^ *VillinCre* with developmental stages of the SI, we used publicly available datasets from ENCODE (ENCSR089FFK, ENCSR842QTB, ENCSR217TMK, ENCSR353IFP, ENCSR211VXF, mm10 genome assembly), removed CpGs which are covered by less than three reads and calculated the mean methylation level for each LMR. Finally, we performed a liftOver^98^ of the *Tet2/3*^*fl/fl*^ and *Tet2/3*^*fl/fl*^ *VillinCre* samples to the mm10 genome assembly, following a combined analysis of the developmental stages with our data.

### Targeted bisulfite DNA methylation analysis

For deep DNA bisulfite sequencing, 100 ng of genomic DNA for each mouse sample was treated with bisulfite, using the EpiTect Bisulfite Kit (Qiagen), according to the manufacturer’s instructions. Treated DNA was amplified with sequence-specific primers (Supp. Table 6) containing Illumina Nextera handle sequence (Forward overhang: 5’TCGTCGGCAGCGTCAGATGTGTATAAGAGACAG‐[locus‐specific sequence] and Reverse overhang: 5’GTCTCGTGGGCTCGGAGATGTGTATAAGAGACAG‐locus-specific sequence]). The primers were selected to amplify a sequence shorter than 200 bp that contained four to nine CpG sites. PCR products of 100–200 bp were gel-extracted using the QIAquick Gel Extraction Kit (Qiagen). Equimolar amounts of all amplicons for each sample were pooled in a single tube. To incorporate the index sequences, the pooled PCR products were amplified by limited cycle-number PCR using Nextera complimentary primers. Each indexed pool was gel-extracted using the QIAquick Gel Extraction Kit, and equimolar amounts of all pools were again pooled in a single tube and processed for sequencing. Sequencing was performed on the pool using MiSeq Reagent Kit v2 (MiSeq, Illumina method). Sequenced reads were separated by barcode, and aligned to the target sequence, and methylation ratios were determined using a Python script (methratio.py).

### RNA extraction, cDNA preparation, and qPCR

Total RNA was extracted from intestinal epithelial cells using miRNeasy (cat. no. 1038703, Qiagen). Reverse transcription was performed using qScript (cat. no. 95047, Quanta Biosciences), and mRNA expression levels were measured with qPCR using SYBR-Green (cat. no. 1725124, Bio-Rad) in a CFX-384 Real-Time PCR system (Bio-Rad). qPCR primers were designed to an exon-exon boundary in all indicated transcripts, when possible (Supp. Table 7). Relative quantities of gene transcripts were normalized to Ubiquitin C (UBC), Hypoxanthine Phosphoribosyltransferase 1 (HPRT) and peptidylprolyl isomerase A (PPIA) transcripts.

### Single-molecule fluorescence in situ hybridization (smFISH)

The smFISH experiments were performed as previously described in refs. ^99,100^. Briefly, the Jejunum part of the small intestine was flushed with ice-cold PBS and then cut open longitudinally. The tissues then were fixed in 4% formaldehyde for 3 h, incubated overnight with 30% sucrose in 4% formaldehyde, and finally embedded in OCT in the form of swiss-rolls. 7 µm thick sections of fixed Jejunum were placed onto poly l-lysine coated coverslips and used for smFISH staining. Sections then were fixed again in 4% formaldehyde in PBS for 15 min at room temperature followed by 70% ethanol dehydration for 2 h at 4 °C. Tissues were treated for 10 min at room temperature with proteinase K (10 μg/ml Ambion AM2546) and washed twice with 2X SSC (Ambion AM9763), then incubated in wash buffer (20% Formamide Ambion AM9342, 2X SSC) for 10 min at room temperature. Next, the tissues were mounted with hybridization buffer (10% Dextran sulfate Sigma D8906, 20% Formamide, 1 mg/ml *E. coli* tRNA, Sigma R1753, 2× SSC, 0.02% BSA, Ambion AM2616, 2 mM Vanadyl-ribonucleoside complex, NEB S1402S) mixed with 1:3000 dilution of specific probe libraries and were transferred to an overnight incubation at 30 °C. After the hybridization, tissues were washed with wash buffer containing 50 ng/ml DAPI for 30 min at 30 °C. smFISH probe libraries for Mmp7 (coupled to Alexa594) and Muc2 (coupled to Cy5) were kindly obtained from Prof. Shalev Itzkovitz’s lab. All images were performed on a Nikon-Ti-2 inverted fluorescence microscope using the NIS element software AR 5.11.01. All images were taken using 100X magnifications.

### Reporting summary

Further information on research design is available in the Nature Portfolio Reporting Summary linked to this article.

## Supplementary information

Supplementary Information

Reporting Summary

## Data Availability

The single-cell RNA-seq, whole-genome bisulfite sequencing (WGBS), and bulk RNA-seq data have been deposited in the National Center for Biotechnology Information (NCBI)’s Gene Expression Omnibus (GEO), and are accessible with accession number GSE200230. The Shotgun metagenomics data have been deposited in the European Nucleotide Archive (ENA) at EMBL-EBI under accession number PRJEB61989. Source data are provided with this paper.

## Source data

Source Data

## References

[1] van der Flier LG, Clevers H (2009). Stem cells, self-renewal, and differentiation in the intestinal epithelium. Annu. Rev. Physiol..

[2] Clevers H (2013). The intestinal crypt, a prototype stem cell compartment. Cell.

[3] Monk M, Boubelik M, Lehnert S (1987). Temporal and regional changes in DNA methylation in the embryonic, extraembryonic and germ cell lineages during mouse embryo development. Development.

[4] Kafri T, Gao X, Razin A (1993). Mechanistic aspects of genome-wide demethylation in the preimplantation mouse embryo. Proc. Natl Acad. Sci. USA.

[5] Kafri T (1992). Developmental pattern of gene-specific DNA methylation in the mouse embryo and germ line. Genes Dev..

[6] Lister R (2009). Human DNA methylomes at base resolution show widespread epigenomic differences. Nature.

[7] Hon GC (2013). Epigenetic memory at embryonic enhancers identified in DNA methylation maps from adult mouse tissues. Nat. Genet..

[8] Ziller MJ (2013). Charting a dynamic DNA methylation landscape of the human genome. Nature.

[9] Tahiliani M (2009). Conversion of 5-methylcytosine to 5-hydroxymethylcytosine in mammalian DNA by MLL partner TET1. Science.

[10] Ko M (2015). TET proteins and 5-methylcytosine oxidation in hematological cancers. Immunol. Rev..

[11] Rasmussen KD, Helin K (2016). Role of TET enzymes in DNA methylation, development, and cancer. Genes Dev..

[12] Gu T (2018). DNMT3A and TET1 cooperate to regulate promoter epigenetic landscapes in mouse embryonic stem cells. Genome Biol..

[13] Orlanski S (2016). Tissue-specific DNA demethylation is required for proper B-cell differentiation and function. Proc. Natl Acad. Sci. USA.

[14] Yue X, Lio CJ, Samaniego-Castruita D, Li X, Rao A (2019). Loss of TET2 and TET3 in regulatory T cells unleashes effector function. Nat. Commun..

[15] Sommer F, Nookaew I, Sommer N, Fogelstrand P, Backhed F (2015). Site-specific programming of the host epithelial transcriptome by the gut microbiota. Genome Biol..

[16] Pan WH (2018). Exposure to the gut microbiota drives distinct methylome and transcriptome changes in intestinal epithelial cells during postnatal development. Genome Med..

[17] Rakoff-Nahoum S (2015). Analysis of gene-environment interactions in postnatal development of the mammalian intestine. Proc. Natl Acad. Sci. USA.

[18] Walker WA (2013). Initial intestinal colonization in the human infant and immune homeostasis. Ann. Nutr. Metab..

[19] Yu DH (2015). Postnatal epigenetic regulation of intestinal stem cells requires DNA methylation and is guided by the microbiome. Genome Biol..

[20] Elliott EN, Sheaffer KL, Schug J, Stappenbeck TS, Kaestner KH (2015). Dnmt1 is essential to maintain progenitors in the perinatal intestinal epithelium. Development.

[21] Sheaffer KL (2014). DNA methylation is required for the control of stem cell differentiation in the small intestine. Genes Dev..

[22] Elliott E. N., Sheaffer K. L. & Kaestner K. H. The ‘de novo’ DNA methyltransferase Dnmt3b compensates the Dnmt1-deficient intestinal epithelium. *Elife* **5**, e12975 (2016). 10.7554/eLife.12975 PMC4786433 26808831

[23] Ansari I (2020). The microbiota programs DNA methylation to control intestinal homeostasis and inflammation. Nat. Microbiol..

[24] Bry L (1994). Paneth cell differentiation in the developing intestine of normal and transgenic mice. Proc. Natl Acad. Sci. USA.

[25] el Marjou F (2004). Tissue-specific and inducible Cre-mediated recombination in the gut epithelium. Genesis.

[26] Becht, E. et al. Dimensionality reduction for visualizing single-cell data using UMAP. *Nat. Biotechnol*. **37**, 38–44 (2018). 10.1038/nbt.4314 30531897

[27] Gehart H (2019). Identification of enteroendocrine regulators by real-time single-. Cell Differ. Mapp. Cell.

[28] Haber AL (2017). A single-cell survey of the small intestinal epithelium. Nature.

[29] Sato T (2011). Paneth cells constitute the niche for Lgr5 stem cells in intestinal crypts. Nature.

[30] Grun D (2016). De novo prediction of stem cell identity using single-cell transcriptome data. Cell Stem Cell.

[31] Jackson DN (2020). Mitochondrial dysfunction during loss of prohibitin 1 triggers Paneth cell defects and ileitis. Gut.

[32] Ghaleb AM, McConnell BB, Kaestner KH, Yang VW (2011). Altered intestinal epithelial homeostasis in mice with intestine-specific deletion of the Kruppel-like factor 4 gene. Dev. Biol..

[33] Ye DZ, Kaestner KH (2009). Foxa1 and Foxa2 control the differentiation of goblet and enteroendocrine L- and D-cells in mice. Gastroenterology.

[34] Gosalia N, Yang R, Kerschner JL, Harris A (2015). FOXA2 regulates a network of genes involved in critical functions of human intestinal epithelial cells. Physiol. Genomics.

[35] Yu T (2012). Kruppel-like factor 4 regulates intestinal epithelial cell morphology and polarity. PLoS ONE.

[36] Schonthaler HB, Guinea-Viniegra J, Wagner EF (2011). Targeting inflammation by modulating the Jun/AP-1 pathway. Ann. Rheum. Dis..

[37] Singh PNP, Madha S, Leiter AB, Shivdasani RA (2022). Cell and chromatin transitions in intestinal stem cell regeneration. Genes Dev..

[38] Jones C, Avino M, Giroux V, Boudreau F (2023). HNF4alpha acts as upstream functional regulator of intestinal Wnt3 and Paneth cell fate. Cell Mol. Gastroenterol. Hepatol..

[39] Lee SH, Veeriah V, Levine F (2022). A potent HNF4alpha agonist reveals that HNF4alpha controls genes important in inflammatory bowel disease and Paneth cells. PLoS ONE.

[40] Lu R (2021). Paneth cell alertness to pathogens maintained by vitamin D receptors. Gastroenterology.

[41] Stine RR (2019). PRDM16 maintains homeostasis of the intestinal epithelium by controlling region-specific metabolism. Cell Stem Cell.

[42] Thakur A (2019). Hepatocyte nuclear factor 4-alpha is essential for the active epigenetic state at enhancers in mouse liver. Hepatology.

[43] Catala-Moll F (2022). Vitamin D receptor, STAT3, and TET2 cooperate to establish tolerogenesis. Cell Rep..

[44] Wilson CL (1999). Regulation of intestinal alpha-defensin activation by the metalloproteinase matrilysin in innate host defense. Science.

[45] Gerbe F (2016). Intestinal epithelial tuft cells initiate type 2 mucosal immunity to helminth parasites. Nature.

[46] Yoon HS (2021). Akkermansia muciniphila secretes a glucagon-like peptide-1-inducing protein that improves glucose homeostasis and ameliorates metabolic disease in mice. Nat. Microbiol..

[47] Ivanov II (2009). Induction of intestinal Th17 cells by segmented filamentous bacteria. Cell.

[48] An J (2015). Acute loss of TET function results in aggressive myeloid cancer in mice. Nat. Commun..

[49] Seritrakul P, Gross JM (2017). Tet-mediated DNA hydroxymethylation regulates retinal neurogenesis by modulating cell-extrinsic signaling pathways. PLoS Genet.

[50] Ji H (2010). Comprehensive methylome map of lineage commitment from haematopoietic progenitors. Nature.

[51] Hodges E (2011). Directional DNA methylation changes and complex intermediate states accompany lineage specificity in the adult hematopoietic compartment. Mol. Cell.

[52] Vella P (2013). Tet proteins connect the O-linked N-acetylglucosamine transferase Ogt to chromatin in embryonic stem cells. Mol. Cell.

[53] Mariappa D, Pathak S, van Aalten DM (2013). A sweet TET-a-tete-synergy of TET proteins and O-GlcNAc transferase in transcription. EMBO J..

[54] Shi FT (2013). Ten-eleven translocation 1 (Tet1) is regulated by O-linked N-acetylglucosamine transferase (Ogt) for target gene repression in mouse embryonic stem cells. J. Biol. Chem..

[55] Charlton J (2020). TETs compete with DNMT3 activity in pluripotent cells at thousands of methylated somatic enhancers. Nat. Genet..

[56] Zhang X (2016). DNMT3A and TET2 compete and cooperate to repress lineage-specific transcription factors in hematopoietic stem cells. Nat. Genet..

[57] Kim R, Sheaffer KL, Choi I, Won KJ, Kaestner KH (2016). Epigenetic regulation of intestinal stem cells by Tet1-mediated DNA hydroxymethylation. Genes Dev..

[58] Sardina JL (2018). Transcription factors drive Tet2-mediated enhancer demethylation to reprogram cell fate. Cell Stem Cell.

[59] Nandan MO (2014). Inducible intestine-specific deletion of Kruppel-like factor 5 is characterized by a regenerative response in adult mouse colon. Dev. Biol..

[60] Golson ML, Kaestner KH (2016). Fox transcription factors: from development to disease. Development.

[61] Gregorieff A (2009). The ets-domain transcription factor Spdef promotes maturation of goblet and paneth cells in the intestinal epithelium. Gastroenterology.

[62] Chen L (2020). HNF4 regulates fatty acid oxidation and is required for renewal of intestinal stem cells in mice. Gastroenterology.

[63] Ali SA (2008). Phenotypic transcription factors epigenetically mediate cell growth control. Proc. Natl Acad. Sci. USA.

[64] Hayashi Y (2014). Downregulation of rRNA transcription triggers cell differentiation. PLoS ONE.

[65] Watanabe-Susaki K (2014). Biosynthesis of ribosomal RNA in nucleoli regulates pluripotency and differentiation ability of pluripotent stem cells. Stem Cells.

[66] Zhang Q, Shalaby NA, Buszczak M (2014). Changes in rRNA transcription influence proliferation and cell fate within a stem cell lineage. Science.

[67] Li HJ (2019). Intestinal Neurod1 expression impairs paneth cell differentiation and promotes enteroendocrine lineage specification. Sci. Rep..

[68] Yin, Y. et al. Impact of cytosine methylation on DNA binding specificities of human transcription factors. *Science* **356**, eaaj2239 (2017). 10.1126/science.aaj2239 PMC8009048 28473536

[69] Ayabe T (2002). Activation of Paneth cell alpha-defensins in mouse small intestine. J. Biol. Chem..

[70] von Moltke J, Ji M, Liang HE, Locksley RM (2016). Tuft-cell-derived IL-25 regulates an intestinal ILC2-epithelial response circuit. Nature.

[71] McGinty JW (2020). Tuft-cell-derived leukotrienes drive rapid anti-helminth immunity in the small intestine but are dispensable for anti-protist immunity. Immunity.

[72] Derrien M, Belzer C, de Vos WM (2017). *Akkermansia muciniphila* and its role in regulating host functions. Micro. Pathog..

[73] Morgan XC (2012). Dysfunction of the intestinal microbiome in inflammatory bowel disease and treatment. Genome Biol..

[74] Fan T. J., Goeser L., Naziripour A., Redinbo M. R. & Hansen J. J. *Enterococcus faecalis* gluconate phosphotransferase system accelerates experimental colitis and bacterial killing by macrophages. *Infect. Immun.* **87**, e00080-19 (2019). 10.1128/IAI.00080-19 PMC6589050 31036600

[75] Vijay-Kumar M (2010). Metabolic syndrome and altered gut microbiota in mice lacking Toll-like receptor 5. Science.

[76] Madison BB (2002). Cis elements of the villin gene control expression in restricted domains of the vertical (crypt) and horizontal (duodenum, cecum) axes of the intestine. J. Biol. Chem..

[77] Cardiff RD, Miller CH, Munn RJ (2014). Manual hematoxylin and eosin staining of mouse tissue sections. Cold Spring Harb. Protoc..

[78] Sato T, Clevers H (2013). Primary mouse small intestinal epithelial cell cultures. Methods Mol. Biol..

[79] Grun D (2015). Single-cell messenger RNA sequencing reveals rare intestinal cell types. Nature.

[80] Stuart T (2019). Comprehensive integration of single-cell data. Cell.

[81] Street K (2018). Slingshot: cell lineage and pseudotime inference for single-cell transcriptomics. BMC Genomics.

[82] Van den Berge K (2020). Trajectory-based differential expression analysis for single-cell sequencing data. Nat. Commun..

[83] Stuart T, Srivastava A, Madad S, Lareau CA, Satija R (2021). Single-cell chromatin state analysis with Signac. Nat. Methods.

[84] Lawrence M (2013). Software for computing and annotating genomic ranges. PLoS Comput. Biol..

[85] Pliner HA (2018). Cicero predicts cis-regulatory DNA interactions from single-cell chromatin accessibility data. Mol. Cell.

[86] Trapnell C, Pachter L, Salzberg SL (2009). TopHat: discovering splice junctions with RNA-Seq. Bioinformatics.

[87] Anders S, Huber W (2010). Differential expression analysis for sequence count data. Genome Biol..

[88] Trapnell C (2010). Transcript assembly and quantification by RNA-Seq reveals unannotated transcripts and isoform switching during cell differentiation. Nat. Biotechnol..

[89] Chen EY (2013). Enrichr: interactive and collaborative HTML5 gene list enrichment analysis tool. BMC Bioinform..

[90] Kuleshov MV (2016). Enrichr: a comprehensive gene set enrichment analysis web server 2016 update. Nucleic Acids Res..

[91] Bolger AM, Lohse M, Usadel B (2014). Trimmomatic: a flexible trimmer for Illumina sequence data. Bioinformatics.

[92] Wood DE, Lu J, Langmead B (2019). Improved metagenomic analysis with Kraken 2. Genome Biol..

[93] Raddatz G, Gao Q, Bender S, Jaenisch R, Lyko F (2012). Dnmt3a protects active chromosome domains against cancer-associated hypomethylation. PLoS Genet..

[94] Xi Y, Li W (2009). BSMAP: whole genome bisulfite sequence MAPping program. BMC Bioinform..

[95] Burger L, Gaidatzis D, Schubeler D, Stadler MB (2013). Identification of active regulatory regions from DNA methylation data. Nucleic Acids Res..

[96] Ernst J, Kellis M (2012). ChromHMM: automating chromatin-state discovery and characterization. Nat. Methods.

[97] Heinz S (2010). Simple combinations of lineage-determining transcription factors prime cis-regulatory elements required for macrophage and B cell identities. Mol. Cell.

[98] Hinrichs AS (2006). The UCSC genome browser database: update 2006. Nucleic Acids Res..

[99] Itzkovitz S (2011). Single-molecule transcript counting of stem-cell markers in the mouse intestine. Nat. Cell Biol..

[100] Moor AE (2018). Spatial reconstruction of single enterocytes uncovers broad zonation along the intestinal villus axis. Cell.

[101] Stahl M (2018). The Muc2 mucin coats murine Paneth cell granules and facilitates their content release and dispersion. Am. J. Physiol. Gastrointest. Liver Physiol..

