# Supplementary material for: TET2 and TET3 loss disrupts small intestine differentiation and homeostasis
Source: Nat Commun. 2023 Jul 6;14:4005. doi: 10.1038/s41467-023-39512-3 (PMC10326054; doi:10.1038/s41467-023-39512-3)
Supplement: Supplementary file 1 — Supplementary Information [file 41467_2023_39512_MOESM1_ESM.pdf]

TET2 and TET3 loss disrupts small intestine differentiation and homeostasis

Ihab Ansari[1], Llorenç Solé-Boldo[2], Meshi Ridnik[1], Julian Gutekunst[2], Oliver Gilliam[2], Maria Korshko[1], Timur Liwinski[3,4], Birgit Jickeli[3], Noa Weinberg-Corem[1], Michal Shoshkes-Carmel[1], Eli Pikarsky[5], Eran Elinav[3,6], Frank Lyko[2] and Yehudit Bergman[1*]

[1]Department of Developmental Biology and Cancer Research, Institute for Medical Research Israel-Canada, Hebrew University Medical School, Jerusalem, Israel; [2]Division of Epigenetics, DKFZ-ZMBH Alliance, German Cancer Research Center, Heidelberg, Germany; [3]Department of Immunology, The Weizmann Institute of Science, Rehovot, Israel; [4]University Psychiatric Clinics Basel, Clinic for Adults, University of Basel, Basel, Switzerland; [5]The Lautenberg Center for Immunology, Institute for Medical Research Israel-Canada, Hebrew University Medical School, Jerusalem, Israel; and [6]Division of Microbiome and Cancer, German Cancer Research Center (DKFZ), Heidelberg, Germany. These authors contributed equally: Ihab Ansari, Llorenç Solé-Boldo.

Corresponding author: Yehudit Bergman

Institute of Medical Research Israel-Canada

Hebrew University Medical School, Jerusalem,

Israel

phone: ++972-2-6758362, fax: ++972-2-6757482

email: Yehudit.bergman@mail.huji.ac.il

Supplementary Table 1. Single-cell RNA-seq sample table.

Sample	Seurat object ID	Cells before QC	Cells after QC	Avg. genes/cell	Avg.UMI/cell
**WT_1**	S1	5,412	3,632	1,759	9,333
**WT_2**	S2	2,497	1,699	1,910	11,256
**WT_3**	S5	7,930	7,691	2,050	9,825
**KO_1**	S3	3,657	2,131	1,868	9,237
**KO_2**	S4	4,751	2,704	1,944	7,702
**KO_3**	S7	8,649	7,836	2,174	8,503

Supplementary Table 2. Bulk RNA-seq sample table.

Sample	Yield	Raw reads	Reads after QC	Mapped reads
**WT1**	6,484,935,090	127,155,590	127,154,362	123,381,563
**WT2**	5,856,100,959	114,825,509	114,824,022	110,627,949
**WT3**	7,563,820,863	148,310,213	148,308,602	143,282,140
**KO1**	6,532,164,762	128,081,662	128,079,998	122,808,962
**KO2**	9,091,189,059	178,258,609	178,256,743	172,422,755
**KO3**	6,624,655,098	129,895,198	129,893,540	122,855,821

Supplementary Table 3. Whole Genome Bisulfite Sequencing (WGBS) sample table.

Sample	Comments	Yield	Raw read pairs	Coverage	Read pairs after QC	Mapped read pairs	Uniquely mapped read pairs	Read pair duplicates	Conversion ratio
**cre-tet11**	Tet2/2 fl/fl	57,854,656,722	191,571,711	5.0 X	184,638,798	146,997,152	NA	14,326,530	99.72%
**cre-tet12**	Tet2/2 fl/fl	66,485,087,392	220,149,296	5.8 X	211,216,953	168,625,882	NA	19,746,919	99.65%
**cre-tet13**	Tet2/2 fl/fl	65,675,779,034	217,469,467	5.8 X	210,697,577	165,747,981	NA	20,319,578	99.73%
**cre-tet14**	Tet2/2 fl/fl Villincre	60,872,480,208	201,564,504	5.7 X	194,605,935	160,657,465	NA	17,161,885	99.74%
**cre-tet15**	Tet2/2 fl/fl Villincre	62,078,586,064	205,558,232	5.7 X	199,544,120	160,742,081	NA	18,026,696	99.70%
**cre-tet16**	Tet2/2 fl/fl Villincre	66,031,970,518	218,648,909	6.0 X	211,694,538	172,999,112	NA	19,880,280	99.76%

Supplementary Table 4. Disease activity index (DAI) score used to evaluate the DSS-induced colitis.

**Score**	**Body weight decrease (%)**	**Stool consistency**	**Rectal bleeding**
**0**	< 1	Normal	Normal
**1**	1–5		
**2**	5–10	Loose stools	
**3**	10–20		
**4**	>20	Diarrhea	Gross bleeding

Supplementary Table 5. Average coverage by read per base for each exon of *Tet2* and *Tet3* genes.

Tet2								
	Start	End	**WT1**	**WT2**	**WT3**	**KO1**	**KO2**	**KO3**
Exon 12	133126640	133131154	19.24	16.865	20.532	17.393	26.64	22.491
Exon 11	133132239	133132603	23.91	20.443	15.305	0.675	0.021	0.005
Exon 10	133134303	133134441	10.612	9.083	14.749	0.337	0	0.023
Exon 9	133136559	133136649	3.546	3.806	6.141	0.586	0.02	0.017
Exon 8	133139448	133139599	16.975	5.116	19.19	14.475	12.005	11.812
Exon 7	133142305	133142329	1.464	0	1.211	0	0	2
Exon 6	133143096	133143305	14.089	2.383	11.616	10.396	13.854	6.422
Exon 5	133144297	133144391	2.251	3.083	6.965	2.953	4.101	1.304
Exon 4	133146202	133146281	3.459	1.183	0.534	0.531	0.482	0.408
Exon 3	133148475	133151681	8.029	5.073	1.691	1.535	4.48	3.673
Exon 2	133176992	133177137	4.16	0.56	0.767	0.532	11.718	0.179
Exon 1	133207163	133207354	0.303	0.172	0.966	0.337	0.023	0.318

Tet3								
	Start	End	**WT1**	**WT2**	**WT3**	**KO1**	**KO2**	**KO3**
Exon 11	83312367	83319819	66.624	63.466	81.149	61.683	88.818	53.725
Exon 10	83320376	83320713	52.091	38.136	65.663	35.022	57.895	26.342
Exon 9	83323187	83323325	35.398	14.07	20.283	7.144	16.382	38.986
Exon 8	83323776	83323866	60.632	25.376	33.373	37.441	60.893	3.204
Exon 7	83325743	83325894	16.084	6.55	15.056	6.198	22.503	5.298
Exon 6	83326759	83326968	28.033	13.853	60.788	3.587	30.855	11.083
Exon 5	83329865	83329959	59.721	2.491	34.226	6.992	6.097	4.409
Exon 4	83335978	83336069	4.241	4.662	18.749	8.106	41.273	12.864
Exon 3	83352660	83354818	61.068	35.829	54.875	7.625	0.357	0.126
Exon 2	83391210	83391267	34.898	14.126	28.323	9.926	19.084	9.868
Exon 1	83391543	83391672	1.606	1.59	1.693	0.996	1.444	0.98

Supplementary Table 6. Primers for targeted bisulfite sequencing.

Gene	LMR position	5' →3'	Primer sequence
Slc16a7	chr10:124707305-124707724	Forward	TTATGAAGGAGTTAAGAGTGATATGAGTAA
		Reverse	AATATTTACTTTTCCCCATCATAAAATT
Pla2g2a	chr4:138387475-138387690	Forward	TTGTAAGTTTTTAGAAGTGATTGTTGG
		Reverse	ATAAACATACCCAATACCCTTTTTATTC
Lyz1	chr10:116729781-116730101	Forward	AGTTGTTGGTTGATTAGAGAAAGTATGT
		Reverse	AAAAAATCAAATACTAAAATCCATAAATCT
Reg4	chr3:98039961-98040630	Forward	TATTGTAGTAAATATTGAGGAGGTTTTTAG
		Reverse	AAAATACCAAAAAAAATTATAAAATAAACA
Pla2g2f	chr4:138310377-138310706	Forward	GGAGGATTAAATATGGTTTATGTTAGTTG
		Reverse	TACTCAAAACTCCAACCTCAATTTACT
Espn	chr4:151502927-151503504	Forward	TTTAGTTGGATTTTTTTTGGGTTTATAT
		Reverse	TTACATACCTTTAAACTTTAATCTTCCC
Plcb2	chr2:118545275-118545556	Forward	TTGTATAATTTTGTGATTTTTAAAAGGA
		Reverse	AAATAATACTATCAACCCAAAAAACTTC
Gfi1b	chr2:28474157-28474446	Forward	AAAGAAAGGGGAATTAATTATTTATAATGT
		Reverse	TACTAAAACCCAAATACCATCACTATCT
Avil	chr10:126438071-126438832	Forward	GATTTTGAAGTTTTATGGGGGATAT
		Reverse	CAAAAAATAATCAACTTTCCTTCAAAC
Pou2f3	chr9:42989689-42990112	Forward	ATTGTTTTAATAAGATGGAAAGTGGAAT
		Reverse	TCCACTATAAATACAAAAACAAATTTCCTA

Supplementary Table 7. Primers used for qPCR analysis.

Gene	5' →3' Forward	5' →3' Reverse
Tet2	CATGTTTGGACTTCTCTGCTCA	CCGACTTCTCGATTGTCTTCTC
Tet3	GGAAATAAATGCTCGTGAAGGA	CTGAGTCCATCTGACCTGGAAC
Ubc	CAGCCGTATATCTTCCCAGACT	CTCAGAGGGATGCCAGTAATCTA
Ppia	CGCGTCTCCTTCGAGCTGTTTG	TGTAAAGTCACCACCCTGGCACAT
Hprt1	GCAGTACAGCCCCAAAATGG	GGTCCTTTTCACCAGCAAGCT
Lyz1	CAGGGTGGTGAGAGATCC	AAGCGAGGAAGTGTGACC
Pla2g2a	GGGCCAAATCACCTGTTCT	GTTCCGGGCGAAACATTC
Pla2g2f	CCAGCCTGGGTATGAAGAAAT	CATGGACTTCAGGTTCAGCA
Reg4	TCCGGAAGCTAAGAAACTGG	TGACACTGGCTTCCTTTTGA
Slc16a7	TCAGATCGCTTGGATATCGTC	GGCTGCCATAGTTATTCACCA
Avil	AGTACCACGAGTCCGACACC	GCAGCCTCTTCACATCATAGG
Espn	GCAGAAGATGCAGGAGGAAG	CGAAGAATGTCTCGTCTCCAG
Gfi1b	AATGCCACGGTCCTTTCTAGT	TTTTGCCACAGGAATTACAGC
Plcb2	TTAGACTGTTGGAAGGGCAAG	GCTTCAATTGCTTCCTTGAAC
Pou2f3	GGAGGATCTGGGTGACTCTCT	GTGGATGGAGAGAAGCCATGT

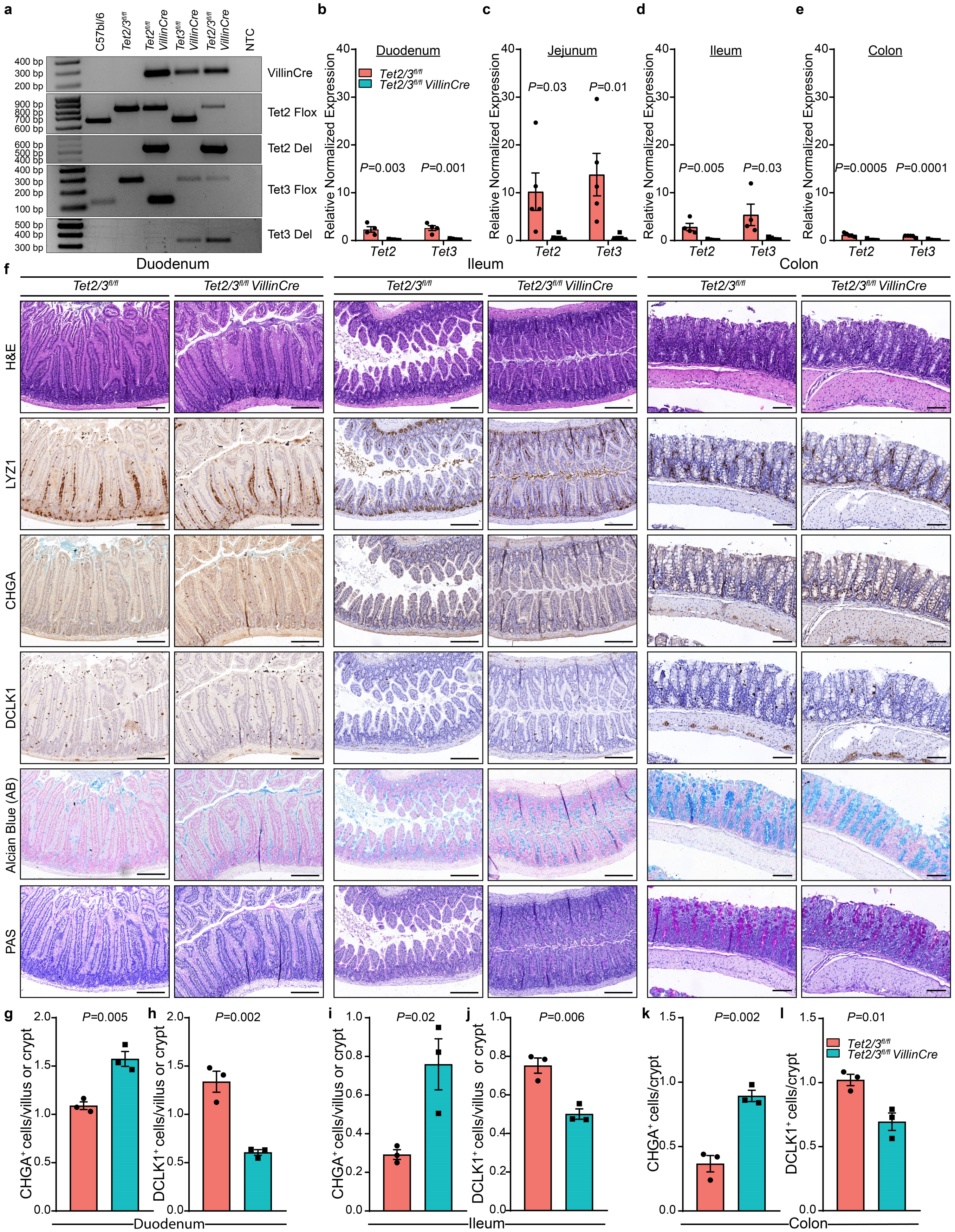
a
C57bl/6
Tet2/3fl/fl
Tet2fl/fl VillinCre
Tet3fl/fl VillinCre
Tet2/3fl/fl VillinCre
NTC
400 bp
300 bp
200 bp
VillinCre
900 bp
800 bp
700 bp
600 bp
Tet2 Flox
600 bp
500 bp
400 bp
Tet2 Del
400 bp
300 bp
200 bp
100 bp
Tet3 Flox
500 bp
400 bp
300 bp
Tet3 Del
b
Duodenum
Tet2/3fl/fl
Tet2/3fl/fl VillinCre
Relative Normalized Expression
P=0.003
P=0.001
Tet2
Tet3
c
Jejunum
P=0.03
P=0.01
d
Ileum
P=0.005
P=0.03
e
Colon
P=0.0005
P=0.0001
f
Duodenum
Ileum
Colon
H&E
LYZ1
CHGA
DCLK1
Alcian Blue (AB)
PAS
g
P=0.005
CHGA+ cells/villus or crypt
h
P=0.002
DCLK1+ cells/villus or crypt
Duodenum
i
P=0.02
j
P=0.006
Ileum
k
P=0.002
CHGA+ cells/crypt
l
P=0.01
DCLK1+ cells/crypt
Colon

Supplementary Figure 1. TET2/3 are needed for SI homeostasis. a, PCR used for genotyping wt and dko transgenic mice. **b**, Normalized expression levels of *Tet2* and *Tet3* genes of duodenal crypts isolated from *Tet2/3$^{fl/fl}$* (wt) (n=4) and *Tet2/3$^{fl/fl}$ VillinCre* (dko) (n=5) mice. **c**, Normalized expression levels of *Tet2* and *Tet3* genes of jejunal crypts isolated from wt (n=5) and dko (n=5) mice. **d**, Normalized expression levels of *Tet2* and *Tet3* genes of ileum crypts isolated from wt (n=4) and dko (n=5) mice. **e**, Normalized expression levels of *Tet2* and *Tet3* genes of colonic crypts isolated from wt (n=5) and dko (n=5) mice. **f**, H&E and Immunohistochemical staining for LYZ1, CHGA, DCLK1 expression and Alcian Blue (AB) and periodic acid schiff stain (PAS) of duodenum, ileum and colon isolated from wt and dko mice. Scale bar 200 µm (Duodenum and ileum) and 100 µm (colon). **g,h**, Quantification of CHGA-positive cells (**g**) and DCLK1-positive cells (**h**) in wt (n=3) and dko (n=3) mice of duodenal region. **i,j**, Quantification of CHGA-positive cells (**i**) and DCLK1-positive cells (**j**) in wt (n=3) and dko (n=3) mice of ileum region. **k-l**, Quantification of CHGA-positive cells (**k**) and DCLK1-positive cells (**l**) in wt (n=3) and dko (n=3) mice of colonic region. Significance (b-e,g-l) was determined using two-sided *t*-test and is expressed as the mean ± SEM. Source data are provided as a Source Data file.

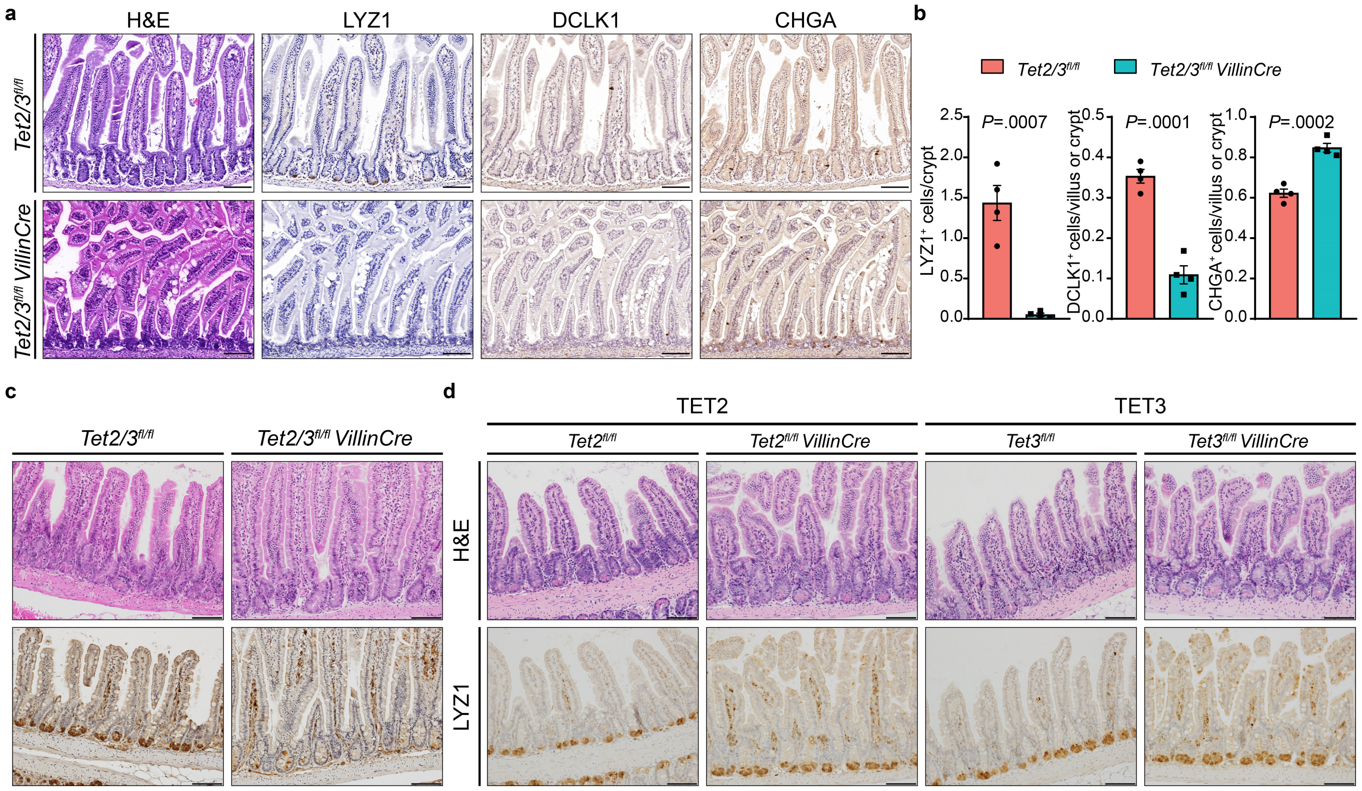

Supplementary Figure 2. Paneth cells are lost in young dko mice. a, H&E and immunohistochemical staining for LYZ1, DCLK1, CHGA proteins of the jejunal region isolated from 14-days-old mixed-sex wt and dko mice. Scale bar 50 µm. **b**, Quantification of LYZ1-, DCLK1- and CHGA- positive cells in 14-day old dko (n=4) mice compared with age-matched wt (n=4) mice. Significance was determined using two-sided *t*-test and is expressed as the mean ± SEM. **c**, H&E and immunohistochemical staining of the jejunum for LYZ1 showed the loss of Paneth cells in dko (n=4) female mice, compared with sex-matched wt (n=4) mice. Scale bar 50 µm. **d**, H&E and immunohistochemical staining of the jejunum for LYZ1 showed mature Paneth cells in TET2 (n=8) and TET3 (n=9) ko mice, compared with wt (n=4) mice. Scale bar 50 µm. Source data are provided as a Source Data file.

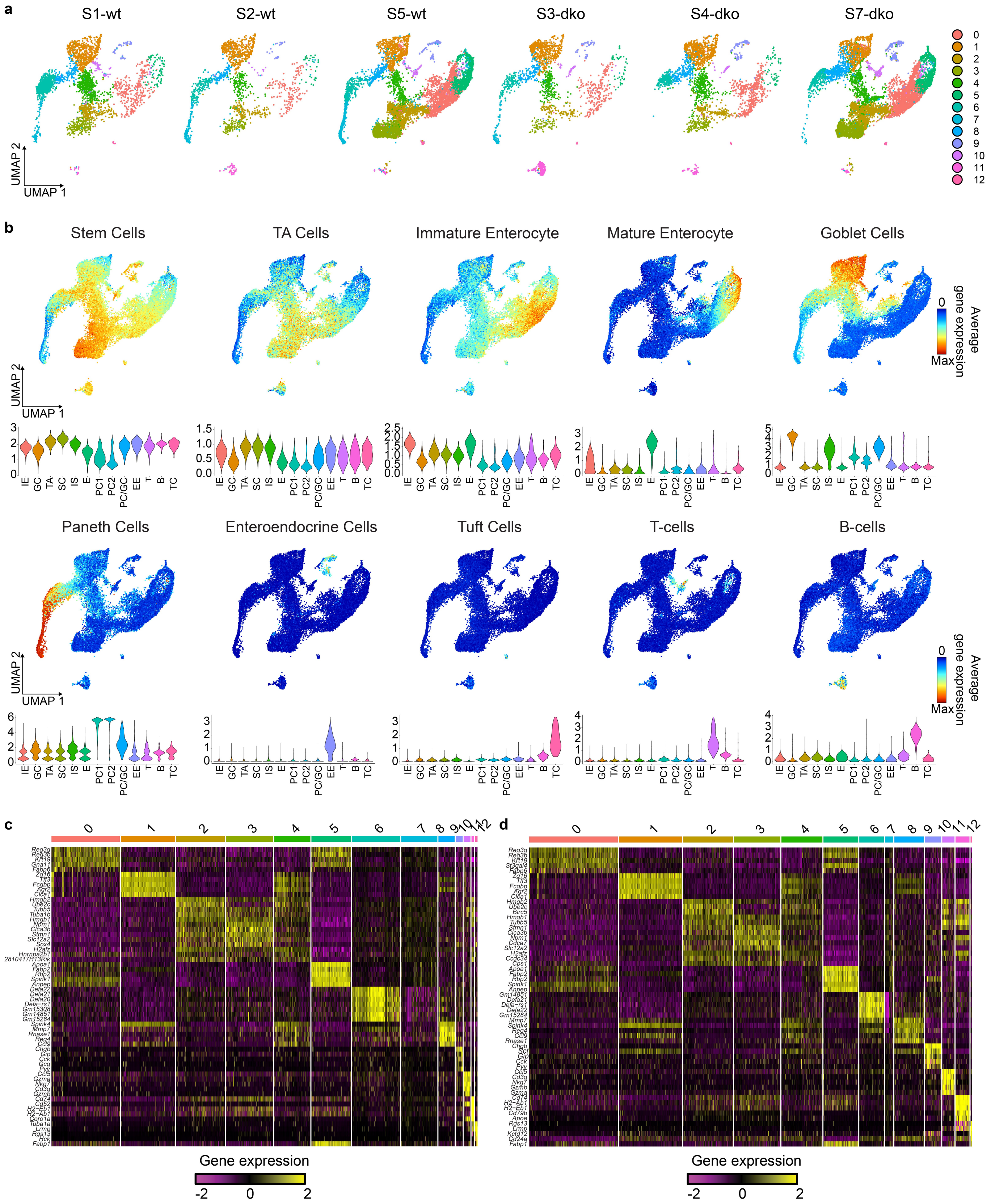
a
S1-wt
S2-wt
S5-wt
S3-dko
S4-dko
S7-dko
UMAP 2
UMAP 1
0
1
2
3
4
5
6
7
8
9
10
11
12
b
Stem Cells
TA Cells
Immature Enterocyte
Mature Enterocyte
Goblet Cells
Average gene expression
0
Max
IE GC TA SC IS E PC1 PC2 PC/GC EE T B TC
Paneth Cells
Enteroendocrine Cells
Tuft Cells
T-cells
B-cells
c
d
Gene expression
-2
0
2

Supplementary Figure 3. Characterization of the intestinal cell populations in wt and dko mice at single-cell resolution. a, UMAP plots showing the contribution of each sample to the distinct cell populations identified in the integrated scRNA-seq analysis (wt=S1, S2 and S5, dko=S3, S4 and S7). **b**, UMAP and violin plots showing the average expression of well-established cell type markers used to identify all cell populations (see Methods for details). Red indicates maximum gene expression, while blue indicates low or no expression of a particular set of genes in log-normalized UMI counts. **c**, Heatmap represents the expression of the top markers for each cell population in wt mice. **d**, Heatmap represents the expression of the top markers for each cell population in dko mice.

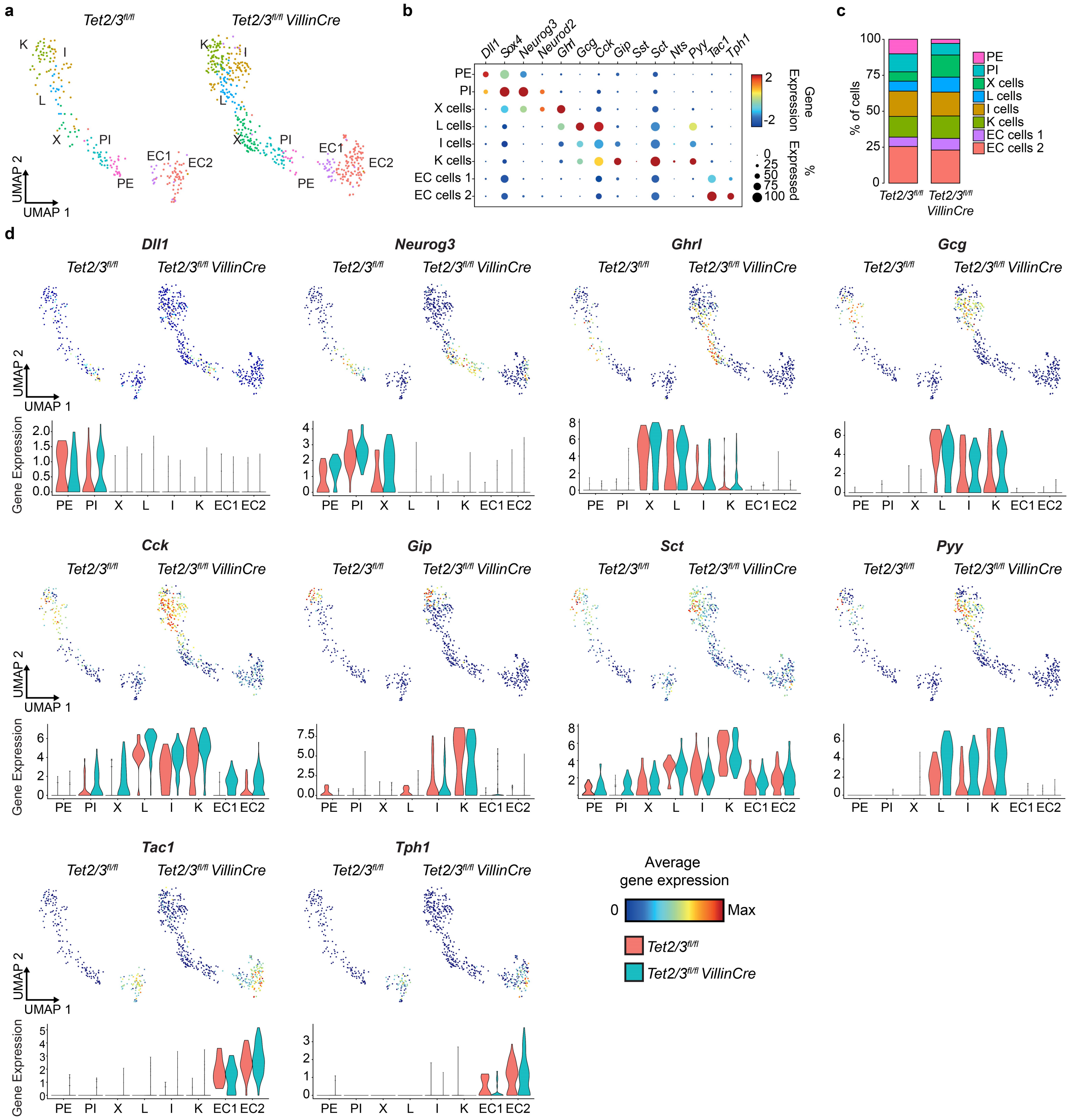

Supplementary Figure 4. Detailed analysis of the Enteroendocrine compartment of wt and dko mice. a, UMAP of wt and dko enteroendocrine cells re-clustered and labelled. **b,** Dot Plot showing the expression of progenitor and hormone genes in all cells. Red indicates high expression while blue indicates low or no expression. Dot size indicated the percentage of cells in each cluster expressing a particular gene. **c,** Barplot showing the subtype composition for the EE compartment in wt and dko mice crypts. **d,** UMAP and Violin plots of progenitors and hormone genes used to identify functional EE cell subtypes. PE: Progenitors early, PI: Progenitors intermediate and EC: Enterochromaffin.

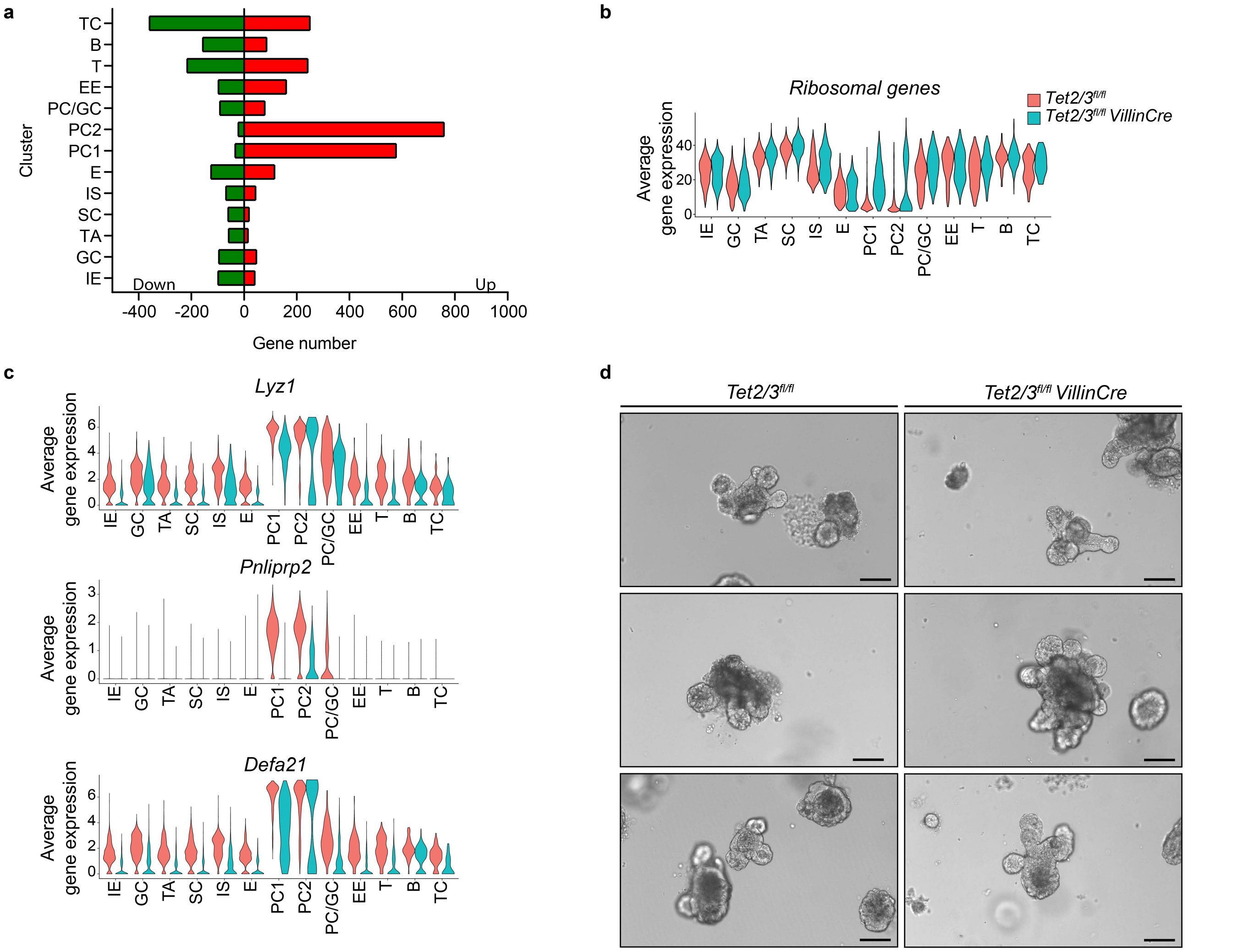

Supplementary Figure 5. TET2/3-depletion effects on PC functions. a, Bar plot showing the number of differentially expressed genes of each cell cluster in the jejunum of wt and dko mice, as provided by Seurat. **b**, Violin plots showing the expression of ribosomal-related genes in each cluster of wt and of dko**. c**, Violin plots showing the expression of Paneth-related genes; *Lyz1*, *Pnliprp2* and *Defa21,* in each cluster of wt and dko. X axes (b and c) depict intestinal clusters and Y axes represent gene expression in log-normalized UMI counts. **d**, Representative images of intestinal organoids derived from wt and dko mice. This experiment was repeated independently three times with the same results. Scale bar 100 μm. Source data are provided as a Source Data file.

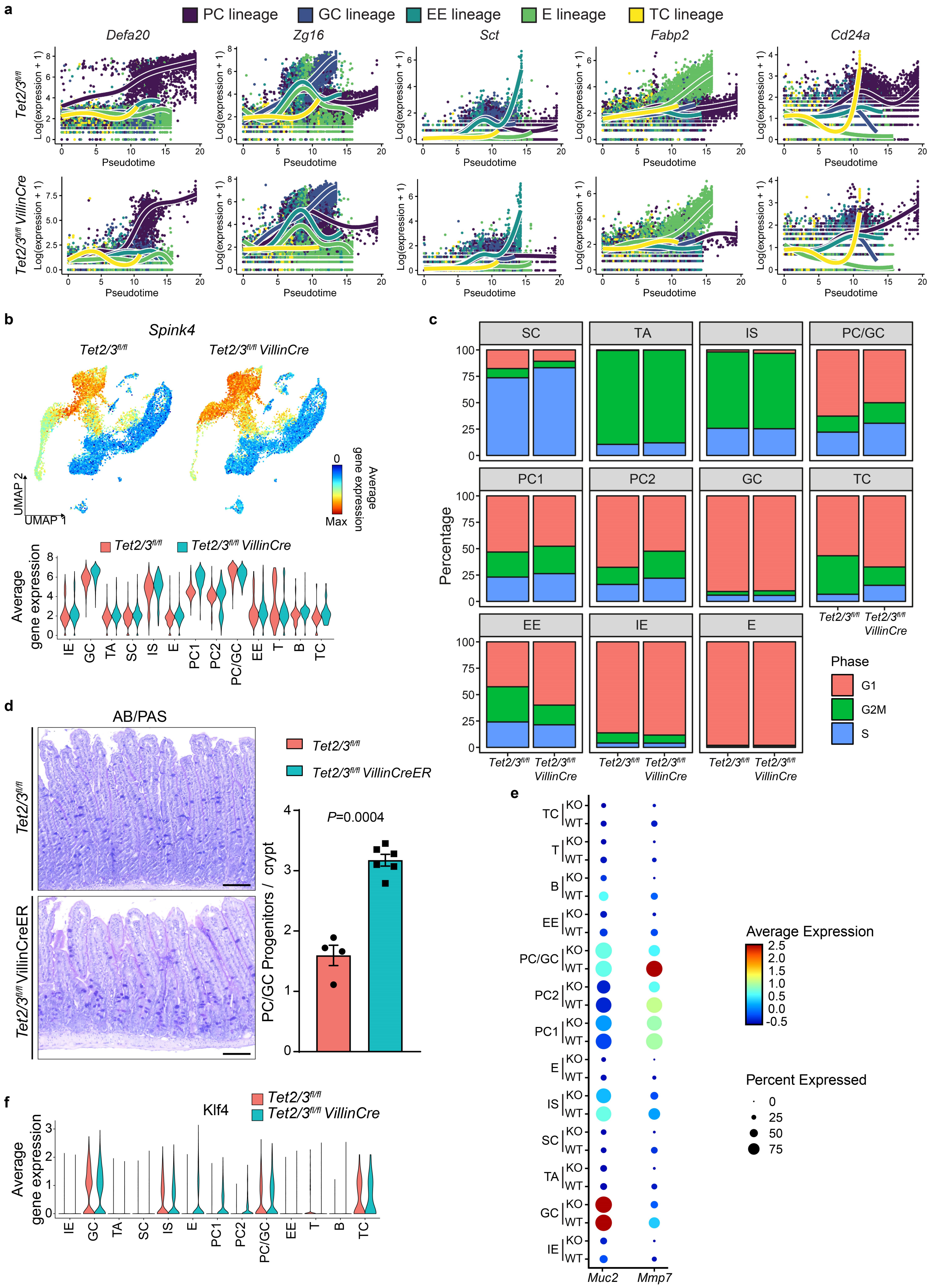
a
PC lineage
GC lineage
EE lineage
E lineage
TC lineage
Defa20
Zg16
Sct
Fabp2
Cd24a
Log(expression + 1)
Pseudotime
b
Spink4
UMAP 2
UMAP 1
Average gene expression
c
SC
TA
IS
PC/GC
PC1
PC2
GC
TC
EE
IE
E
Percentage
Phase
G1
G2M
S
d
AB/PAS
P=0.0004
PC/GC Progenitors / crypt
e
Average Expression
Percent Expressed
Muc2
Mmp7
f
Klf4

Supplementary Figure 6. Impaired Paneth cell differentiation in TET2/3 dko mice. a, Expression of well-known lineage-specific genes in both wt and dko samples is shown. **b**, UMAP and violin plots showing the expression of Paneth/Goblet progenitors related gene *Spink4* in each cluster of wt and dko. In UMAP gene expression projections, red indicates maximum gene expression while blue indicates low or no expression in log-normalized UMI counts. In violin plots, x-axis depicts intestinal cluster subpopulations and y-axis represents gene expression in log-normalized UMI counts. **c**, Barplots displaying the proportion of PC/GC cells in S, G1 or G2M phases, estimated based on their single-cells transcriptomes. **d,** Alcian Blue/PAS staining of jejunum specimens from $Tet2/3^{fl/fl}$ (n=4) and $Tet2/3^{fl/fl}$ *VillinCreER* (n=6) mice. Quantitation of PC/GC progenitors in the crypt regions. At least 100 crypts were counted from each mouse of each genotype. Scale bar 100 µm. Significance was determined using two-sided *t*-test and is expressed as the mean ± SEM. **e,** Dot Plot showing the expression of *Mmp7* and *Muc2* in all cell types. Red indicates high expression while blue indicates low or no expression. Dot size indicates the percentage of cells in each cluster expressing a particular gene. **f,** Violin plots showing the expression of *Klf4* gene in each cluster of wt and dko. Source data are provided as a Source Data file.

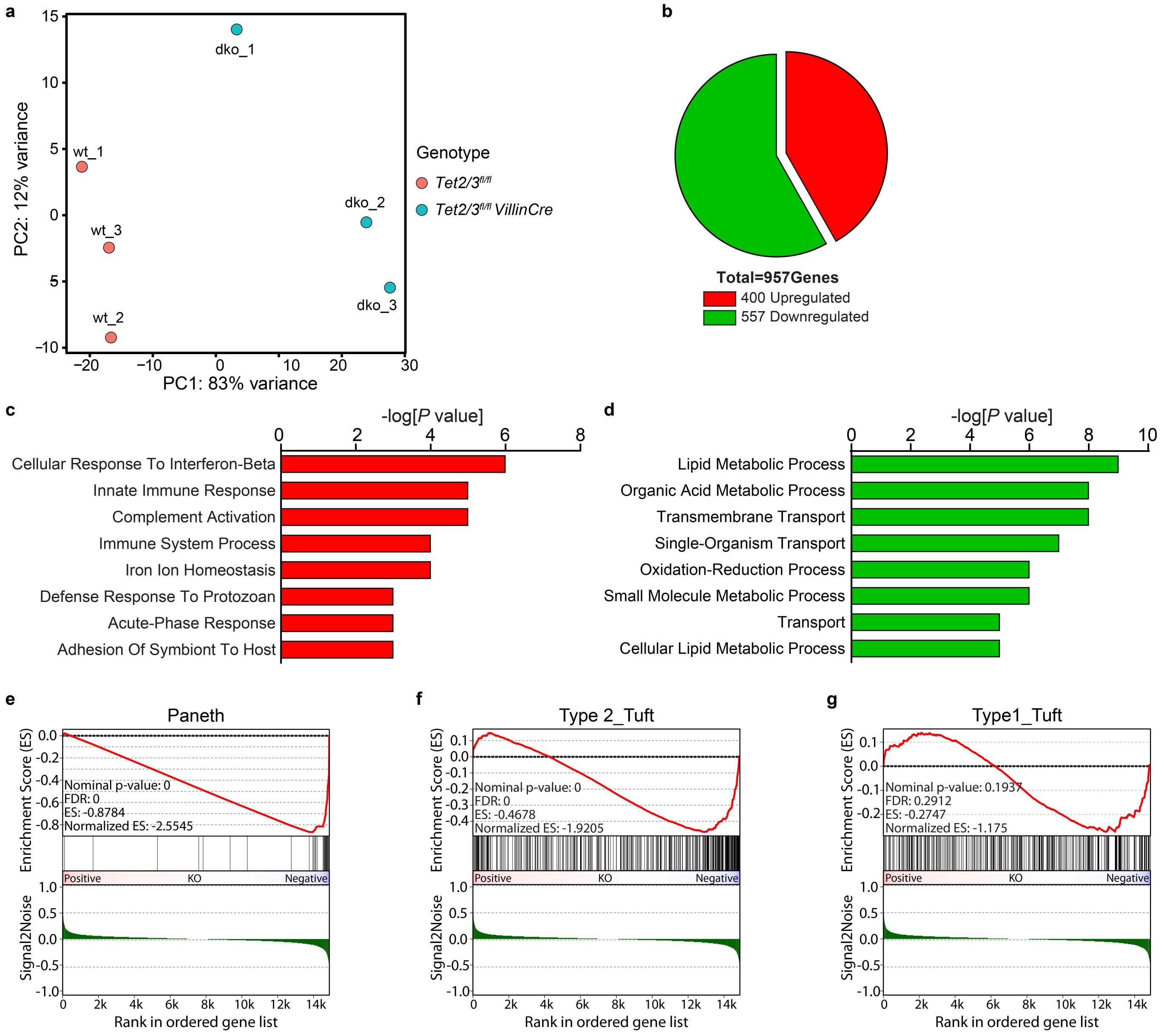

Supplementary Figure 7. Transcriptional changes induced by TET2/3-depletion. a, Principal component analysis (PCA) of crypt bulk transcriptomes clearly separates wt (n=3) and dko (n=3) samples. **b**, A pie chart showing the number of significantly differentially expressed genes in dko (957) with a fold-change of ≥2, relative to wt mice. Upregulated genes in dko mice are shown in red, and downregulated in green. **c**, GO analysis of the 400 upregulated genes from (b). The highly enriched biological processes from the enriched categories are shown. **d**, GO analysis of the 557 downregulated genes from (b). The highly enriched biological processes are shown. *P*-values (c, d) were calculated using two-tailed Fisher's exact test. **e-g**, GSEA analysis based on bulk RNA-seq data for intestinal cells from wt and dko mice. Enrichment is shown for transcriptional signatures related to (**e**) Paneth cells, (**f**) Type 2 Tuft cells and (**g**) Type 1 Tuft cells. Statistics were generated in accordance with the published GSEA algorithm. *p* values (e-g) were calculated based on two-sided Kolmogorov–Smirnov test and adjusted by Benjamini–Hochberg method.

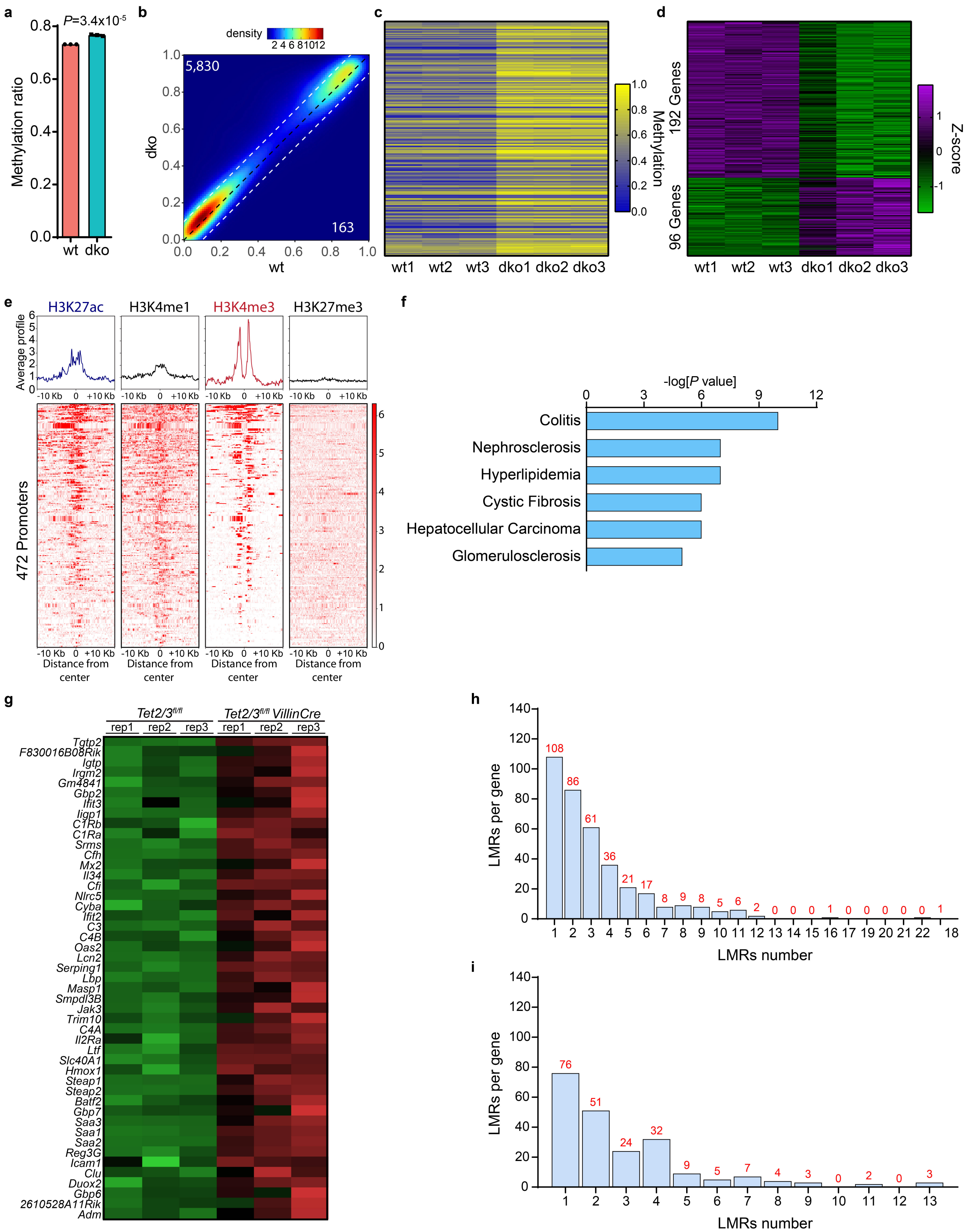
a
P=3.4x10-5
Methylation ratio
wt dko
b
density
2 4 6 8 10 12
5,830
163
dko
wt
c
wt1 wt2 wt3 dko1 dko2 dko3
Methylation
d
192 Genes
96 Genes
Z-score
wt1 wt2 wt3 dko1 dko2 dko3
e
H3K27ac
H3K4me1
H3K4me3
H3K27me3
Average profile
-10 Kb 0 +10 Kb
472 Promoters
Distance from center
f
-log[P value]
Colitis
Nephrosclerosis
Hyperlipidemia
Cystic Fibrosis
Hepatocellular Carcinoma
Glomerulosclerosis
g
Tet2/3fl/fl
Tet2/3fl/fl VillinCre
rep1 rep2 rep3 rep1 rep2 rep3
Tgtp2
F830016B08Rik
Igtp
Irgm2
Gm4841
Gbp2
Ifit3
Iigp1
C1Rb
C1Ra
Srms
Cfh
Mx2
Il34
Cfi
Nlrc5
Cyba
Ifit2
C3
C4B
Oas2
Lcn2
Serping1
Lbp
Masp1
Smpdl3B
Jak3
Trim10
C4A
Il2Ra
Ltf
Slc40A1
Hmox1
Steap1
Steap2
Batf2
Gbp7
Saa3
Saa1
Saa2
Reg3G
Icam1
Clu
Duox2
Gbp6
2610528A11Rik
Adm
h
LMRs per gene
LMRs number
i
LMRs per gene
LMRs number

Supplementary Figure 8. DNA methylation changes in TET2/3 dko mice. a, Average global DNA methylation ratios are shown for wt (n=3) and dko samples (n=3), respectively. Significance was determined using two-sided *t*-test and is expressed as the mean ± SEM. **b**, Comparison of average promoter methylation levels in wt and dko mice. The upper and lower dotted lines indicate the positions in the plot where dko is exactly 0.1 hypermethylated and 0.1 hypomethylated compared to wt, respectively. There are 5,830 (163) promoters which are more than 0.1 hypermethylated (hypomethylated) in dko *vs*. wt samples. **c**, Heatmap of 472 promoters that are associated with gene expression changes in dko samples compared with wt samples. **d**, Heatmap of 192 downregulated and 96 upregulated genes that are associated with hypermethylated promoters in dko samples compared with wt samples. Z-scores computed for genes that are differentially expressed (p adj < 0.05, |log 2 (foldchange)| > 1) between wt and dko samples. **e**, Average histone modification profile of hypermethylated promoters. The normalized signal of different histone modifications measured in a window of ±10,000 bp. **f**, Diseases related of the downregulated genes (n=192) associated with hypermethylated promoters in dko compared to wt samples (*P* values were calculated using two-tailed Fisher's exact test). **g**, Heatmap of inflammatory upregulated genes in dko samples compared with wt samples. Z-scores computed for genes that are differentially expressed (p adj < 0.05, |log 2 (foldchange)| > 1) between wt and dko samples. These genes were obtained from (figure 5f). **h**, Distributions of the 622 hypermethylated LMRs number over the 216 upregulated genes. X-axis stands for the number of LMRs per gene, and Y-axis stands for the numbers of genes in each bin. **i**, Distributions of the 1,192 hypermethylated LMRs number over the 369 downregulated genes. X-axis stands for the number of LMRs per gene, and Y-axis stands for the numbers of genes in each bin. Source data are provided as a Source Data file.

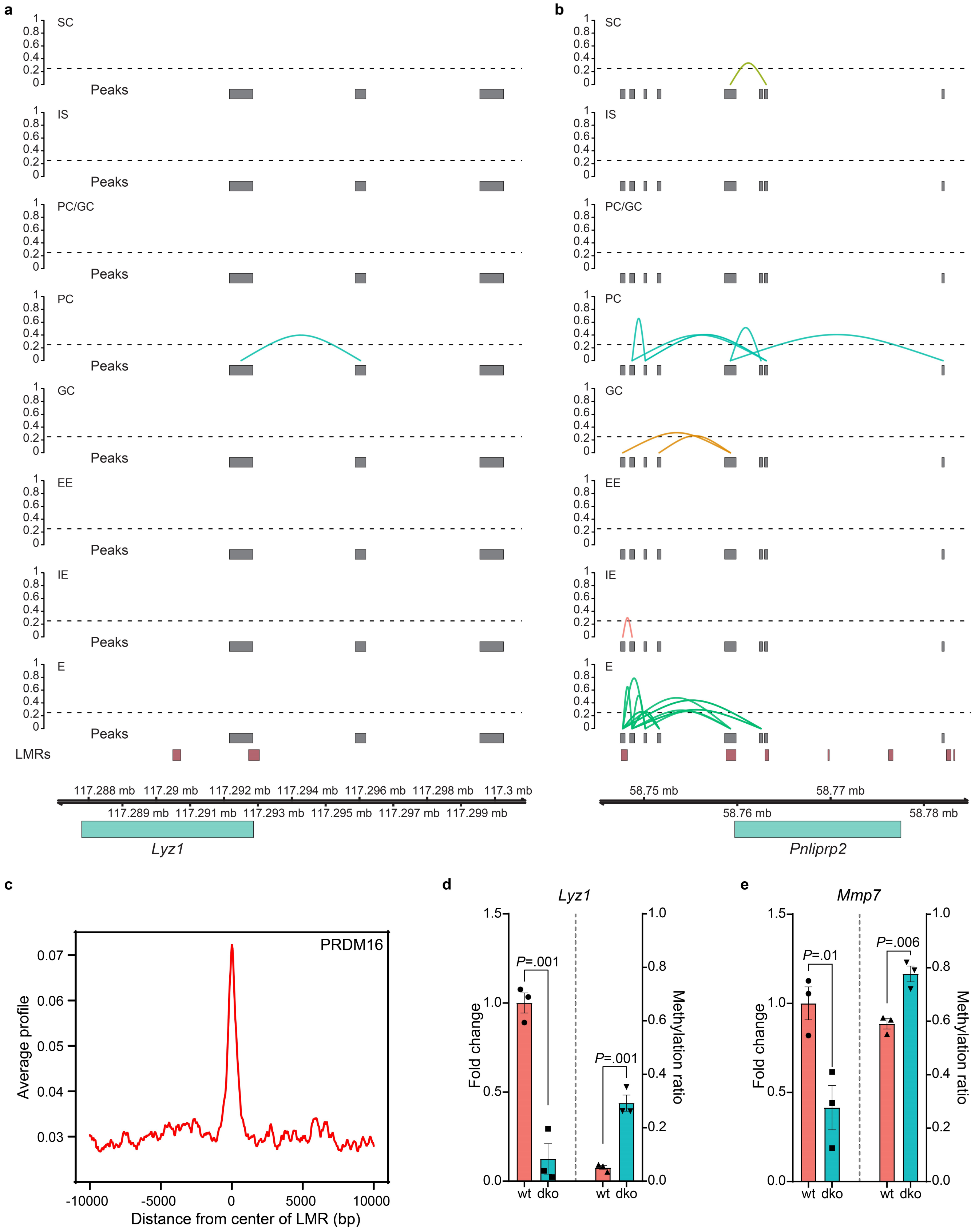

a
SC
IS
PC/GC
PC
GC
EE
IE
E
Peaks
LMRs
117.288 mb 117.29 mb 117.292 mb 117.294 mb 117.296 mb 117.298 mb 117.3 mb
117.289 mb 117.291 mb 117.293 mb 117.295 mb 117.297 mb 117.299 mb
Lyz1
b
58.75 mb 58.77 mb
58.76 mb 58.78 mb
Pnliprp2
c
PRDM16
Average profile
Distance from center of LMR (bp)
d
Lyz1
Fold change
Methylation ratio
P=.001
P=.001
wt dko wt dko
e
Mmp7
P=.01
P=.006

Supplementary Figure 9. Chromatin co-accessibility at Paneth cell-specific genes. a,b, Cicero co-accessibility at Paneth cell-specific (**a**) Lyz1 and (**b**) Pnliprp2 genes calculated for each cell type independently using jejunum crypts from wt mice. Grey colored boxes indicate scATAC-seq peaks and red colored boxes indicate hypermethylated LMRs identified in dko mice. Co-accessibility is represented by arcs connecting peaks. Only connections with a co-accessibility score higher than 0.25 are shown. **c,** Average profile of the transcription factor PRDM16 over the 943 hypermethylated LMRs. The normalized signal of different transcription factors measured in a window of ±10,000 bp. **d,** Bar plot comparing the LMR methylation level and fold change of gene expression level of *Lyz1* gene between wt (n=3) and dko (n=3) samples. Information extracted from WGBS and RNA-seq data. **e**, Bar plot comparing the promoter methylation level and fold change in gene expression of *Mmp7* gene between wt (n=3) and dko (n=3) samples extracted from WGBS and RNA-seq data. Significance (d, e) was determined using two-sided *t*-test and is expressed as the mean ± SEM. Source data are provided as a Source Data file.

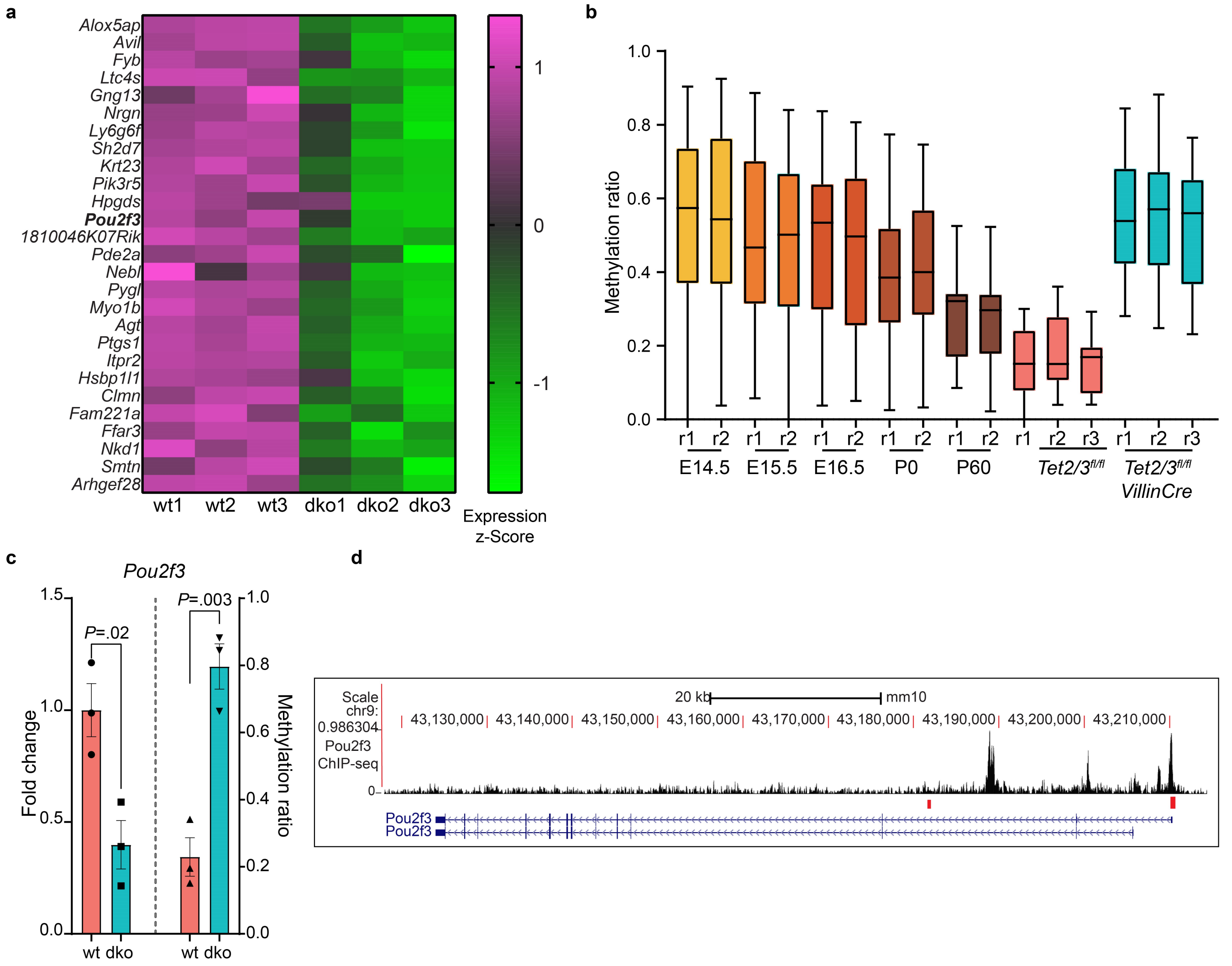

Supplementary Figure 10. Tuft cell-specific LMRs are associated with downregulated gene expression. a, Heatmap of several downregulated Tuft cell markers that contain hypermethylated LMRs in dko samples, compared with wt samples. **b**, Boxplot showing the average methylation level of the hypermethylated LMRs that are associated with Tuft-specific genes during the indicated developmental time points. Two replicates (r1 and r2) are shown for each analyzed sample. Boxplot shows the median, the 25th and 75th percentiles, and the smallest and largest values within 1.5× the interquartile range (whiskers). **c**, Bar plot comparing the LMR methylation level and fold change of gene expression of *Pou2f3* gene between wt (n=3) and dko (n=3) samples, extracted from WGBS and RNA-seq data. Significance was determined using two-sided *t*-test and is expressed as the mean ± SEM. **d**, Displayed is a ~100 kbp genomic region up- and down- stream of the *Pou2f3* gene in the UCSC genome browser (mm10) and ChIP-seq signals obtained with antibody directed against the POU2F3 transcription factor. Red colored boxes represent hypermethylated LMRs in dko mice. Source data are provided as a Source Data file.

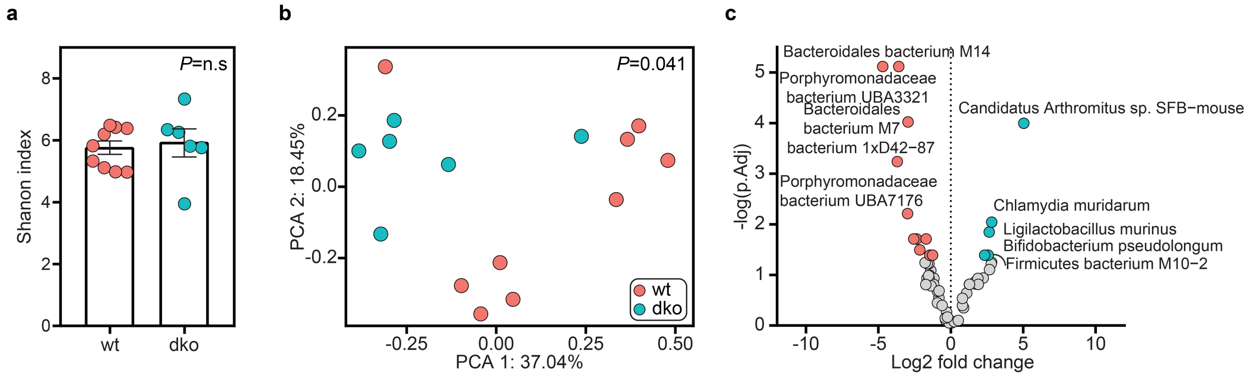

Supplementary Figure 11. Intestinal content microbiota is altered in TET2/3 dko mice. a, Alpha diversity measured by Shannon diversity index in wt (n=9) and dko (n=6) mice. The larger Shannon index value indicates higher community diversity. Significance was determined using two-sided Wilcoxon test and is expressed as the mean ± SEM. **b,** PCA plots of beta diversity analysis of wt (n=9) and dko (n=6) mice. *P*-values were calculated using PERMANOVA test. **c,** Volcano plot showing the significantly enriched (right) and depleted (left) bacteria in dko (n=6) compared with wt (n=9). Significance was determined using two-sided Wald test with Benjamini–Hochberg correction.
