# Supplementary material for: TET2 and TET3 loss disrupts small intestine differentiation and homeostasis
Source: Nat Commun. 2023 Jul 6;14:4005. doi: 10.1038/s41467-023-39512-3 (PMC10326054; doi:10.1038/s41467-023-39512-3)
Supplement: Supplementary file 2 — Reporting Summary [file 41467_2023_39512_MOESM2_ESM.pdf]

nature portfolio

Corresponding author(s): Yehudit Bergman

Last updated by author(s): May 22, 2023

Reporting Summary

Nature Portfolio wishes to improve the reproducibility of the work that we publish. This form provides structure for consistency and transparency in reporting. For further information on Nature Portfolio policies, see our Editorial Policies and the Editorial Policy Checklist.

Statistics

For all statistical analyses, confirm that the following items are present in the figure legend, table legend, main text, or Methods section.

n/a	Confirmed	
☐	☒	The exact sample size (*n*) for each experimental group/condition, given as a discrete number and unit of measurement
☐	☒	A statement on whether measurements were taken from distinct samples or whether the same sample was measured repeatedly
☐	☒	The statistical test(s) used AND whether they are one- or two-sided<br>*Only common tests should be described solely by name; describe more complex techniques in the Methods section.*
☐	☒	A description of all covariates tested
☐	☒	A description of any assumptions or corrections, such as tests of normality and adjustment for multiple comparisons
☐	☒	A full description of the statistical parameters including central tendency (e.g. means) or other basic estimates (e.g. regression coefficient) AND variation (e.g. standard deviation) or associated estimates of uncertainty (e.g. confidence intervals)
☐	☒	For null hypothesis testing, the test statistic (e.g. *F*, *t*, *r*) with confidence intervals, effect sizes, degrees of freedom and *P* value noted<br>*Give P values as exact values whenever suitable.*
☒	☐	For Bayesian analysis, information on the choice of priors and Markov chain Monte Carlo settings
☒	☐	For hierarchical and complex designs, identification of the appropriate level for tests and full reporting of outcomes
☒	☐	Estimates of effect sizes (e.g. Cohen's *d*, Pearson's *r*), indicating how they were calculated

Our web collection on statistics for biologists contains articles on many of the points above.

Software and code

Policy information about availability of computer code

Data collection	Miseq v2, Illumina<br>HiSeq 2000 v4, Illumina<br>NextSeq 550, Ilumina (75 cycles)<br>CFX384 Touch Real-Time PCR Detection System
Data analysis	Bio-Rad CFX Maestro 1.0 (v 4.0.2325.0418)<br>Graphpad Prism version 8.0<br>BSMAP 2.5<br>Picard tool (http://broadinstitute.github.io/picard)<br>methratio.py<br>Tophat 2.0.6<br>Cuffdiff 2.0<br>EnrichR (Disease_Perturbations_from_GEO_down, GO_Biological_Process_2018)<br>MACS2 (version 2.1.1.20160309)<br>R studio (v3.4.3-v3.6.3)<br>Microsoft Excel (Microsoft Office, 2016)<br>HOMER 4.9.1<br>Signac (v.1.8.0)<br>Seurat (v.3.1.1 & v.4.2.0)<br>slingshot (v1.6.1)<br>TradeSeq (v1.5.06)

GenomicRanges package (v.1.48.0)
Cicero (v.1.3.8)
DESeq 2.(v1.10.1-v1.12.3) (https://bioconductor.org/packages/release/bioc/html/DESeq2.html)
Fastp (0.20.1) (https://github.com/OpenGene/fastp)
Bowtie2 2.2.4 (https://github.com/BenLangmead/bowtie2)
Bracken 2.6.2 (https://github.com/jenniferlu717/Bracken)
Seqtk (v.1.2-r94-v.1.3-r114) (https://github.com/lh3/seqtk)
Kraken2 2.1.2 (https://github.com/DerrickWood/kraken2)
KneadData (v0.7.2)
DIAMOND (v.2.0.15)
EMPANADA (https://github.com/borenstein-lab/empanada)
vegan (https://rdrr.io/cran/vegan/man/vegan-package.html)
mixOmics (http://mixomics.org/)
MethylSeekR (https://bioconductor.org/packages/release/bioc/html/MethylSeekR.html)

For manuscripts utilizing custom algorithms or software that are central to the research but not yet described in published literature, software must be made available to editors and reviewers. We strongly encourage code deposition in a community repository (e.g. GitHub). See the Nature Portfolio guidelines for submitting code & software for further information.

Data

Policy information about availability of data

All manuscripts must include a data availability statement. This statement should provide the following information, where applicable:

- Accession codes, unique identifiers, or web links for publicly available datasets
- A description of any restrictions on data availability
- For clinical datasets or third party data, please ensure that the statement adheres to our policy

The single-cell RNA-seq, whole genome bisulfite sequencing (WGBS), and bulk RNA-seq data have been deposited in the National Center for Biotechnology Information (NCBI)'s Gene Expression Omnibus (GEO), and are accessible through the GEO Series accession number, GSE200230, including GSE200227, GSE200228 and GSE200229. The Shotgun metagenomics data have been deposited in the European Nucleotide Archive (ENA) at EMBL-EBI under accession number PRJEB61989. Source data are provided with this paper.

Human research participants

Policy information about studies involving human research participants and Sex and Gender in Research.

Reporting on sex and gender	N/A
Population characteristics	N/A
Recruitment	N/A
Ethics oversight	N/A

Note that full information on the approval of the study protocol must also be provided in the manuscript.

Field-specific reporting

Please select the one below that is the best fit for your research. If you are not sure, read the appropriate sections before making your selection.

☒ Life sciences ☐ Behavioural & social sciences ☐ Ecological, evolutionary & environmental sciences

For a reference copy of the document with all sections, see nature.com/documents/nr-reporting-summary-flat.pdf

Life sciences study design

All studies must disclose on these points even when the disclosure is negative.

Sample size	No sample-size calculation was performed. We chose the sample-size in each experiment based on our previous studies (DOI: 10.1038/s41564-019-0659-3, DOI: 10.1038/s42003-020-0922-4)<br>For in vivo experiments, we performed at least 3-5 independent replicates for each.<br>We used sample sizes commonly accepted for high throughput genome-wide experiments. We performed 3 biological replicates for WGBS. The results of the WGBS were validated using at least 5 independent replicates by targeted bisulfite sequencing.
Data exclusions	No samples were excluded in this study.
Replication	Each experiment was replicated n times (and n is given in each figure for each experiment). Although the exact quantitative results differ

Replication	between replicates, the qualitative results were the same, so that it is reasonable to state that the "All attempts at replication were successful".
Randomization	The mice were allocated randomly in this study
Blinding	The data collection was not blinded. Blinding was not possible as the investigators were also conducting the experiments and had to be aware of controls and treated groups. Pathology analysis was single-blinded.

Reporting for specific materials, systems and methods

We require information from authors about some types of materials, experimental systems and methods used in many studies. Here, indicate whether each material, system or method listed is relevant to your study. If you are not sure if a list item applies to your research, read the appropriate section before selecting a response.

Materials & experimental systems

n/a	Involved in the study
☐	☒ Antibodies
☒	☐ Eukaryotic cell lines
☒	☐ Palaeontology and archaeology
☐	☒ Animals and other organisms
☒	☐ Clinical data
☒	☐ Dual use research of concern

Methods

n/a	Involved in the study
☒	☐ ChIP-seq
☒	☐ Flow cytometry
☒	☐ MRI-based neuroimaging

Antibodies

Antibodies used	- Ki-67 Monoclonal Antibody (cat. no. MA5-14520; dilution 1:200; Thermo Fisher; clone SP6; Lot#: QH2066534)<br>- LYZ1 Monoclonal Antibody (cat. no. ab108508; dilution 1:1000; Abcam, clone: EPR2994(2), Lot#: GR3256655-1)<br>- DCLK1 Monoclonal Antibody (cat. no. 62257; dilution 1:300; CellSignaling, clone: D2U3L, Lot#:1)<br>-CHGA Polyclonal Antibody (cat. no. ab15160; dilution 1:400; Abcam, Lot#: GR3229573-3)<br>-OLFM4 Monoclonal Antibody (cat. no. 39141; dilution 1:400; CellSignaling, clone:)D6Y5A, Lot#: 1)<br>- ImmPRESS® HRP Horse Anti-Rabbit IgG Polymer Detection Kit, Peroxidase was used as secondary antibodies (Vector Laboratories, Cat# MP-7401,Lot#: ZF0906)
Validation	All antibodies used in this study were validated by the manufacturer.<br>- Ki-67 Monoclonal Antibody: (https://www.thermofisher.com/antibody/product/Ki-67-Antibody-clone-SP6-Monoclonal/MA5-14520)<br>- LYZ1 Monoclonal Antibody: (https://www.abcam.com/products/primary-antibodies/lysozyme-antibody-epr29942-ab108508.html)<br>- DCLK1 Monoclonal Antibody: (https://www.cellsignal.com/products/primary-antibodies/dclk1-dcamkl1-d2u3l-xp-rabbit-mab/62257)<br>- CHGA Polyclonal Antibody: (https://www.abcam.com/products/primary-antibodies/chromogranin-a-antibody-ab15160.html)<br>- OLFM4 Monoclonal Antibody: (https://www.cellsignal.com/products/primary-antibodies/olfm4-d6y5a-xp-rabbit-mab-mouse-specific/39141)

Animals and other research organisms

Policy information about studies involving animals; ARRIVE guidelines recommended for reporting animal research, and Sex and Gender in Research

Laboratory animals	All mice are C57BL/6 or have been backcrossed to the C57BL/6 background.<br>- We crossed Tet2/3fl/fl with VillinCre mice to generate Tet2/3fl/fl and Tet2/3fl/fl VillinCre mice. Mice between 8-10 weeks were used in this study.<br>- We also generated TET2/3-inducible mutant mice by crossing Tet2/3fl/fl with VillinCreER mice to generate Tet2/3fl/fl and Tet2/3fl/fl VillinCreER mice. Mice at age of postnatal 1 day (P1) were injected with tamoxifen and then were sacrificed at age of 4 weeks.
Wild animals	The study did not involve wild animals.
Reporting on sex	Both males and females were used in this study.
Field-collected samples	This study did not involve samples collected from the field.
Ethics oversight	The Animal Care and Use Committee of The Hebrew University of Jerusalem approved all animal procedures.

Note that full information on the approval of the study protocol must also be provided in the manuscript.
